# Assessing water requirements and suitability for apple growth at county scale in China: a phenological modeling approach during key growth stages

**DOI:** 10.3389/fpls.2025.1572647

**Published:** 2025-05-12

**Authors:** Xiaoya Ru, Tianzhi He, Guochao Yan, Yong He, Zhujun Zhu, Qiang Yu, Jianqiang He

**Affiliations:** ^1^ College of Horticulture Science, Zhejiang A&F University, Hangzhou, Zhejiang, China; ^2^ Key Laboratory for Agricultural Soil and Water Engineering in Arid Area of Ministry of Education, Northwest A&F University, Yangling, Shaanxi, China; ^3^ Key Laboratory of Quality and safety Control for Subtropical Fruit and vegetable, Ministry of Agriculture and Rural Affairs, Hangzhou, Zhejiang, China; ^4^ State Key Laboratory of Soil Erosion and Dryland Farming on the Loess Plateau, Institute of Water and Soil Conservation, Northwest A&F University, Yangling, Shaanxi, China

**Keywords:** apple, phenology model, irrigation water requirements, evapotranspiration, water suitability

## Abstract

Water shortages greatly challenge high-quality apple production in dryland agricultural regions. Bridging the gap between water use and apple crop water requirements, as well as clarifying water suitability levels, are essential steps to improve water use efficiency. This study innovatively introduced phenological models to accurately predict apples’ phenological stages, thus constructing a dynamic crop coefficient (K_c_) curve. By skillfully integrating this curve with classic FAO 56 Penman-Monteith (P-M) ET_o_ model, the water requirements (WR) and water suitability (S) were evaluated during apple flowering-fruit setting, fruit expansion, and coloring-maturity stages. The results showed that the average durations of the apple phenological stages were 22 days for flowering-fruit setting, 102 days for fruit expansion, and 39 days for fruit coloring-maturity. Unexpectedly, counterintuitive results emerged regarding water requirements and suitability across the phenophases. Despite the fruit expansion stage having the highest average water requirement (319 mm), multi-year data indicated ‘relatively suitable’ (S=0.8) conditions for most counties. In contrast, although the average water requirement during flowering-fruit setting was 120 mm, the suitability level was classified as ‘unsuitable’ (S=0.3), indicating a water shortage, particularly in Xinjiang, the northwest Loess Plateau, and northern Bohai Bay areas. The coloring-maturation stage, with an average water requirement of 113 mm, was classified as ‘very suitable’ (S=1.5), reflecting highly favorable conditions. As this stage progressed, over-humidity conditions began in the Southwestern Cool Highlands and spread to the southwestern Loess Plateau. These findings revealed that the relationship between water requirements and suitability was not linear and emphasized the critical need for focused water management during the flowering-fruit setting stage to ensure sustainable apple production.

## 1 Introduction

Apple trees, as perennial crops with high water requirements, consumed substantial amounts of water to produce high-quality fruit during various phenological stages (Wang and Liu, 2022). China was one of the world’s largest apple producers, with the highest output and an extensive production area. Most high-quality apples with commercial value were grown in dryland agricultural regions, such as the Yellow River Basin, Haihe River Basin, and Aksu River Basin (Sugiura et al., 2023; Vujadinović Mandić et al., 2023). Irrigation systems built near these river basins ensured the sustainable development of local fruit production (Wang et al., 2020a). However, the accelerating impacts of climate change increasingly highlighted the instability of irrigation water resources for orchards in these areas. This raised serious concerns for apple production and highlighted the need to optimize water resource management strategies to mitigate the potential crisis and ensure the stable growth of apple cultivation.

Recently, methods needed to improve management of actual water use by crops in irrigated agriculture have been developed providing useful insights on orchard water use patterns. Among them, soil-based methods, which use soil moisture sensors to monitor water conditions in the root zone and surrounding areas, and plant-based methods, which rely on plants as biosensors to integrate soil and climatic conditions along with physiological responses to water deficits, are widely used (Juillion et al., 2022; Wang et al., 2020b). Despite their usefulness, studies conducted using these methods in fields and small catchments have generally failed to provide comprehensive spatial water characteristics of soil and orchards at a regional scale, limiting their applicability for large-scale water management (Dzikiti et al., 2018). Moreover, most existing studies tend to focus only on a specific growth stage of the orchard (Jia et al., 2020; Wang et al., 2021a; Zanotelli et al., 2019), which leaves a significant gap in detailed, continuous, quantitative data regarding how water requirements fluctuate from flowering to fruit maturity.

In addition to these methods, the concept of water suitability has become increasingly important in agricultural water management and has proven effective in guiding water management planning across extensive crop areas. The level of water suitability measures how closely local effective rainfall aligns with the ideal water conditions required for crop growth. For instance, Elnashar et al. (2021) assessed the water suitability of pome fruits using the ALES-Arid, Ref-ET, and SEBAL models, while Fadl and Abuzaid (2017) calculated the water requirements of various crops, including peach, citrus, olive, and sunflower using the FAO-Cropwat model. These researches demonstrated that the concept of water suitability was widely applicable to various crops. Similarly, Gao et al. (2018) quantified apple water suitability in the Loess Plateau of China using the Penman-Monteith equation, revealing important trends in regional water availability for apple production. However, while water suitability index has been effectively applied, there remains a research gap in quantifying water suitability for fruit trees, particularly apples, across multiple phenological stages. Therefore, comprehensive studies are needed to better understand how water suitability evolves during the different phenological stages of apples, from flowering to fruit maturation.

Water requirements calculation was the core foundation of water suitability. The most widely used estimate of water requirements for a particular crop is obtained multiplying the reference evapotranspiration (ET_o_, the sum of evaporation and transpiration of homogeneously clipped and well-irrigated grass field (Allen et al., 1998; Monteith, 1965; Penman, 1948), calculated by the FAO 56 Penman-Monteith (P-M) ET_o_ equation, by a crop coefficient (K_c_), yielding ET_c_=ET_o_×K_c_. ET_o_ is determined based on meteorological data from reference stations. However, K_c_ represents an integration of the primary characteristics that differentiate the grass reference from the crop regarding energy balance (Allen et al., 1998). Therefore, the K_c_ value must be adjusted for specific crops, tree spacing, regional climates, and canopy coverage at different growth stages (Doorenbos et al., 1977; Panigrahi, 2023; Ye et al., 2020). In practice, there are two primary approaches to obtain K_c_ values: one relies on field observations, while the other predicts K_c_ values based on observed surface cover conditions and height ratios (Dang et al., 2020; Hublart et al., 2016). Both methods presented significant challenges for woody crops like fruit trees or crops with incomplete canopy cover. The unique growth habits and complex canopy structure of fruit trees made field observations difficult, while prediction methods based on ground cover could not capture the full growth details, leading to significant data discrepancies (Liu et al., 2023). To address these challenges, the application of phenological models became crucial. Phenological models could simulate the dynamic growth of apple trees across different phenological stages, helping to understand their changes over time (Fernandez et al., 2022; Yaacoubi et al., 2019). By using these models, more accurate Kc-time curves could be constructed, overcoming the limitations of traditional methods and providing an effective tool for optimizing water management strategies in apple orchards and improving the precision of Kc estimation.

The objectives of this study are to estimate the water requirement of apple trees at the county scale in China’s main apple-producing areas, and to assess water suitability levels during apple three phenological stages. Specifically, the study focused on: (1) Quantifying phenological stages: The time window of three key phenological stages of apple development—flowering-fruit setting, fruit expansion, and fruit coloring-maturity—were identified and quantified using an ensemble of multiple phenological models; (2) Estimating water requirements: the FAO 56 Penman-Monteith (P-M) ET_o_ equations, combined with crop coefficients (K_c_) specific to different phenological stages, were applied to estimate potential evapotranspiration (ET_o_) and subsequently calculate the water requirements for apple trees; (3) Introducing the index of water suitability level: The water suitability level for apples was quantified by considering the spatiotemporal evolution characteristics of effective precipitation during the three phenological stages. The study provided a scientific basis for the irrigation planning and management of apple orchards.

## 2 Materials and methods

### 2.1 Study area

**Figure 1:**
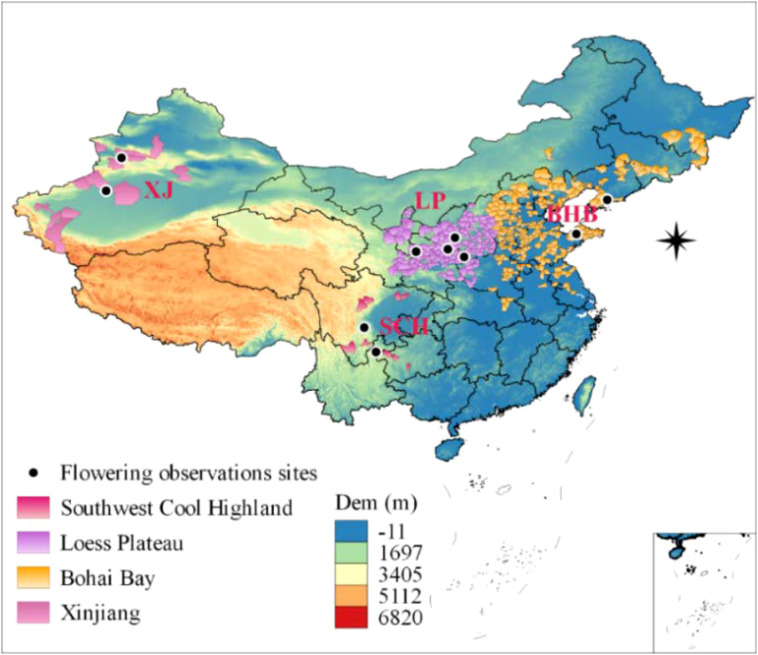
Map of four main apple cultivation regions in China’s: the Loess Plateau region (LP), the Bohai Bay region (BHB), the Southwest Cool Highland region (SCH), and the Xinjiang region (XJ). The black dots are the location information of the apple flowering dates observation sites.

China’s apple cultivation covers vast regions and can be divided into four main apple-producing areas (**Figure 1**): the Loess Plateau region, the Bohai Bay region, the Southwest Cool Highland region, and the Xinjiang region (Zhang et al., 2020). The sum of apple planting area in the Loess Plateau and Bohai Bay regions amounts to 1.63 million hectares, yielding a total apple production of 37.3 million tons, which constitutes 82.4% and 87.9% of China’s total apple orchard area and production, respectively Wang et al., 2019). The total apple production of Southwest Cool Highland approximately 2 million tons in 2021 (Lijia and Xuexi, 2014). “Fuji” is dominant apple variety, with planting area and total production exceeding 50% and 76.4%, respectively (Lu et al., 2022).

### 2.2 Data sources

#### 2.2.1 Meteorological data

The meteorological data, which comprise two weather datasets, were collected from China Meteorological Data Sharing Network (http://data.cma.cn). This first dataset includes daily measured R_s_ and other accessible weather variable measurements from 80 solar radiation observation stations in China (**Supplementary Table S1**). The second dataset contains both benchmark and basic meteorological stations across 367 primary apple-growing counties from 1960 to 2020. The accessible weather variables in the two datasets include maximum temperature (*T_max_*, °C), minimum temperature (*T_min_*, °C), precipitation (*P*, mm), wind speed (*U*, m s ^− 1^), relative humidity (*RH*, %), and sunshine hours (*n*, h). In addition, the high costs of establishment and maintenance of solar radiation observation equipment have resulted in a limited number of solar radiation monitoring stations in China, restricting the application of the Penman-Monteith model. To address this limitation, the previous research performed quality control on meteorological data and utilized machine learning combined with the Ångstrom-Prescott equation to estimate solar radiation at 839 meteorological stations based on the solar radiation observation (Chen et al., 2022; He et al., 2020).

#### 2.2.2 Phenological data

The observation data of apple flower dates were obtained from the Shaanxi Meteorological Bureau (http://sn.cma.gov.cn/) and the Nanjing Shuxi Intelligent Technology Co., Ltd. (http://www.shuxitech.com/). The dataset for flowering dates spanned from 1972 to 2020, encompassing a total of 10 observation counties distributed across the four major apple-producing areas in China. Detailed latitude and longitude information of these flowering observations for each site is provided in **Figure 1** (represented by black dots) and **Table 1**.

**Table 1:** Location details of flowering observations sites.

Apple cultivation regions	County name	Latitude	Longitude	Period
Loess Plateau	Xifeng	35.61	107.03	1984–2018
	Luochuan	35.81	109.67	1972–2018
	Yuncheng	35.17	111.02	2016–2020
	Yanchuan	36.77	110.26	2016–2020
Bohai Bay	Zhuanghe	39.92	122.90	2016–2020
	Laixi	37.08	120.34	2016–2020
Southwest Cool Highland	Zhaotong	27.27	103.70	2016–2020
	Weining	27.07	103.85	2016–2020
Xinjiang	Akesu	43.41	82.56	2016–2020
	Alaer	40.67	81.28	2016–2020

### 2.3 Identifying and modeling apple phenology

#### 2.3.1 Identifying and dividing phenology stage of apples

The critical phenological stages that determined yield and fruit quality in apple trees occurred between flowering and maturity (Han et al., 2023; Yang et al., 2021). We divided this period into three phenological windows: the flowering-fruit setting stage, the fruit expansion stage, and the fruit coloring-maturity stage. Descriptive diagrams of these specific phenological stages of apples and the typical observed occurrence times of phenology are presented in **Table 2**. The three phenological stages of apples mapped to the FAO56 growth stages (initial, mid-season, and late-season). We accurately simulate site-specific flowering dates and subsequently determined the duration from the initial occurrence to the end of these three phenological stages. For instance, we simulated that the flowering date of a particular site is April 3 (93 DOY), and the fruit-setting date is May 3 (123 DOY), indicating a 30-day flowering-fruit setting stage. This practice provided an appropriate framework to construct K_c_ curves on a daily scale.

**Table 2:** Description and illustration of the three time windows of apple growth stages—flowering-fruit setting (Initial Stage in FAO56), fruit expansion (Mid-Season Stage in FAO56), and fruit coloring-maturity (Late-Season Stage in FAO56).

Phenogical stages	Description	Illustration	DOY or dates of apple representative sites					
			LS		BB		SCH	XJ
			YC	LC	BJ	QD	ZT	AKS
Flowering-fruit setting stage	Flowering beginning: 15% of flowers open	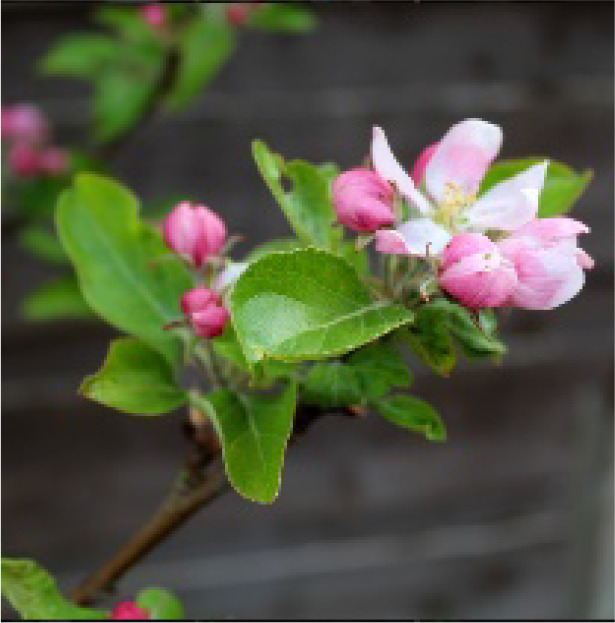	94 Apr 3	105 Apr 14	111 Apr 20	109 Apr 18	81 Mar 21	111 Apr 20
	Ovary growth: fructification visible and all petals fallen or dry.	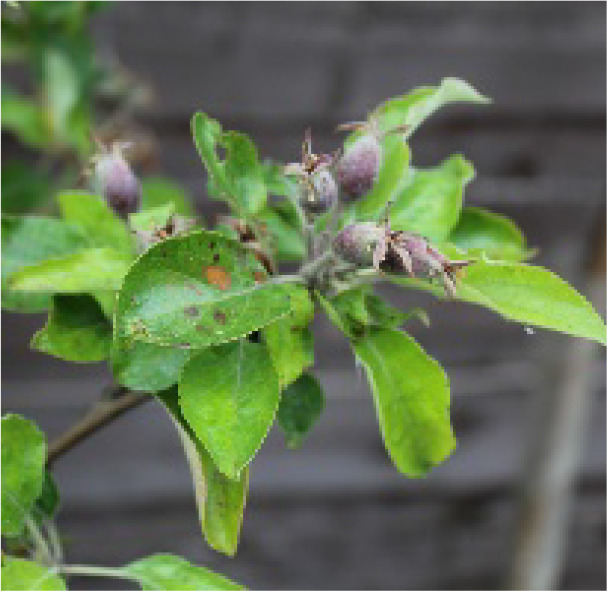	119 Apr 28	124 May 3	128 May 7	131 May 10	109 Apr 18	136 May 15
Fruit expansion stage	Fruits elongation: fruits approximately 20% of final size.	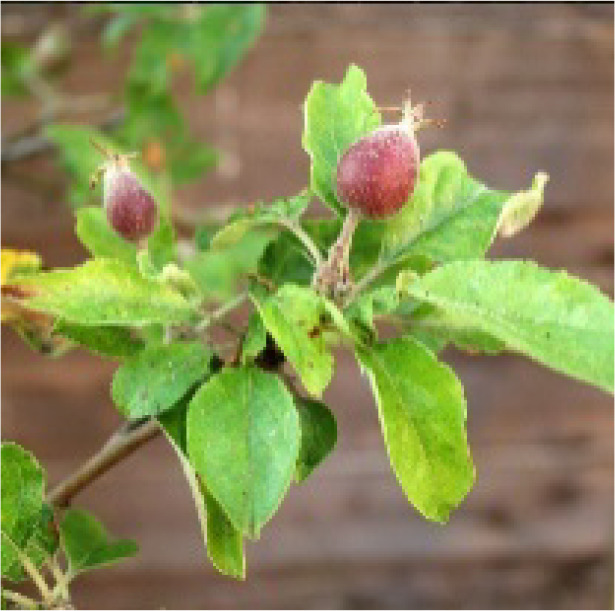	120 Apr 29	125 May 4	129 May 8	132 May 9	110 Apr 19	137 May 16
	Fruits elongation: fruits approximately 80% of final size.	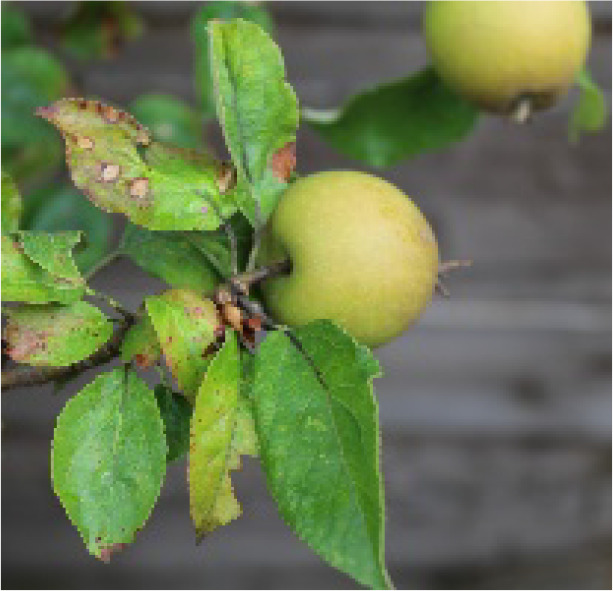	232 Aug 19	239 Aug 26	232 Aug 19	232 Aug 19	192 Jul 10	244 Aug 31
Fruit coloring-maturity stage	Fruit color appears: carpel separation beginning.	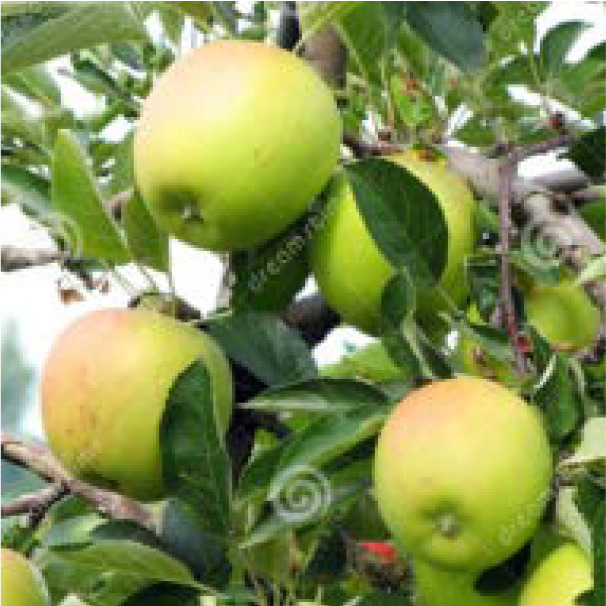	233 Aug 20	240 Aug 27	233 Aug 20	233 Aug 20	193 Jul 10	245 Sep 1
	Fruits ripe and detach easily: physiological maturity.	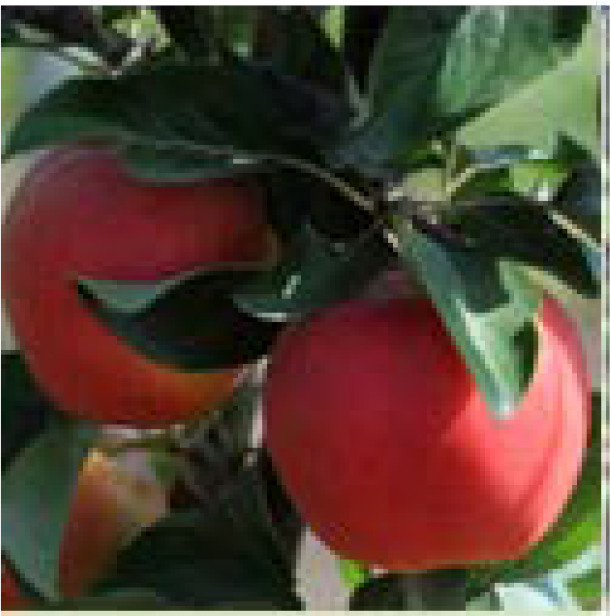	291 Oct 17	284 Oct 10	289 Oct 15	283 Oct 9	259 Sep 15	301 Oct 27

The table also includes observed phenological dates (expressed in DOY or specific dates) across representative base counties in major apple-producing regions of China, including the Loess Plateau (LP), Bohai Bay (BB), Southwest Cool Highland (SCH), and Xinjiang (XJ). The representative base counties were randomly selected from each major apple-producing region, namely Yuncheng (YC), Luochuan (LC), Beijing (BJ), Zhaotong (ZT), and Akesu (AKS) counties.

#### 2.3.2 Apple crop coefficient (K_c_)

**Figure 2:**
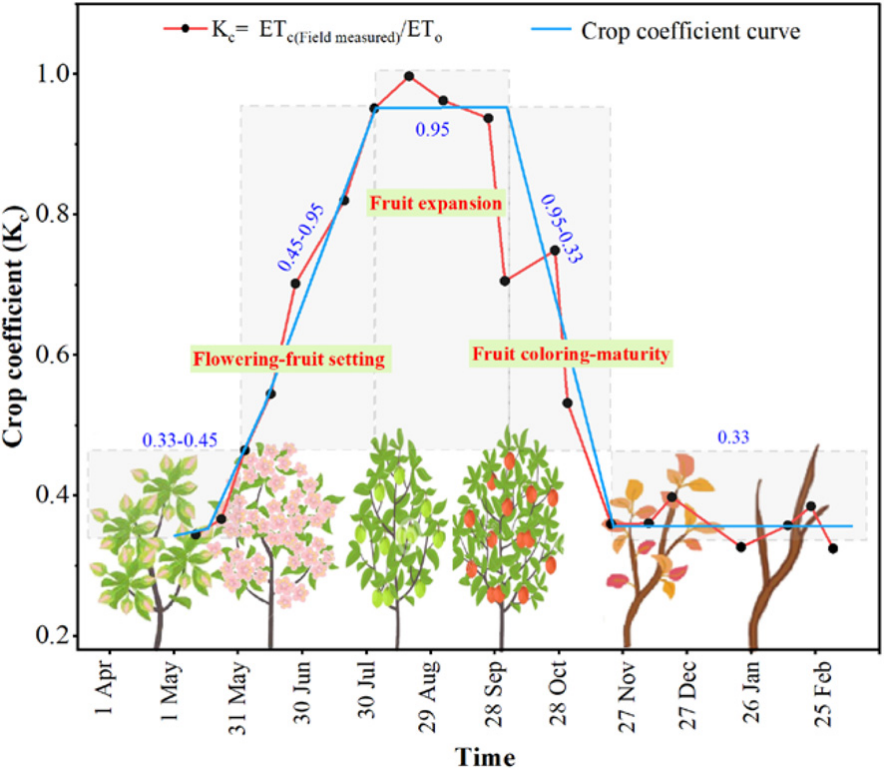
Corrected crop coefficient (K_c_) curves during different phenological stages in the representative apple orchard of China.

The crop coefficient (K_c_) was a dynamic parameter that reflected variations in crop water demand across different phenological stages. Long-term site monitoring in major apple-producing regions of China, including Yan’an (Shaanxi) (Meng, 2011), Linfen (Shanxi) (Dang et al., 2020; Wang et al., 2021a), and the hilly areas of Shandong (Chen, 2011; Liu et al., 2024), revealed characteristic changes in K_c_ for apple trees. Specifically, K_c_ increased from 0.33 to 0.45 during the early phenological stage, rose further to 0.95 during the flowering and fruiting stage, remained stable during the peak stage of fruit expansion, gradually decreased during the coloring-maturity stage, and eventually fell back to 0.33 during the leaf fall and dormancy stage (**Figure 2**). This dynamic pattern corresponded closely to the water requirements of each phenological stage.

In this study, key phenological stages of apple trees—flowering-fruit setting, fruit expansion, and fruit coloring-maturity—were simulated and integrated with the dynamic K_c_ curve. The FAO-56 Penman-Monteith formula was then employed to estimate crop evapotranspiration (ET_c_) for each stage. For instance, K_c_ started at 0.75 during the flowering-fruit setting stage, increased gradually to 0.95 during the fruit expansion stage, remained at 0.95 throughout fruit coloring-maturity stage. These K_c_ values, derived from field measurements under local climatic conditions and apple-specific growth patterns, were well-suited for estimating water requirements.

#### 2.3.3 Apple phenology model

The apple phenological model is based on the temperature response theory of ‘heat forcing’ and ‘chilling requirement’ (Chuine et al., 1999; Ettinger et al., 2020). ‘Heat forcing’ describes the accumulation of heat that stimulates apple trees after dormancy. Once enough heat is accumulated, the apple trees begin flowering. Common forcing response functions include linear, sigmoidal (S-shaped), and exponential functions (Harrington and Gould, 2015). ‘Chilling requirement’ is another important factor in the dormancy release of apple trees. Moderate sub-zero temperatures induce dormancy, which is typically broken after prolonged exposure to chilling but non-freezing temperatures between 2 and 7°C (Basler, 2016; Hänninen, 1987; Luedeling et al., 2009). In current models, the response to chilling temperatures is implemented either as a triangular function of temperature or bell shaped curve (Wang et al., 2020b).

This study used a weighted ensemble model of four phenological models to simulate the flowering date of apple. These four models are the Photothermal-time model (M1) (Blümel and Chmielewski, 2012), the Uniforc model (Chuine, 2000), the Alternating model (Benmoussa et al., 2018; Chuine, 2000), and the Unichill model (**Table 3**) (Caffarra et al., 2011). The first two models solely account for ‘heat forcing’, whereas the latter two models integrate sub-models of ‘chilling requirements’ and ‘heat forcing’ in different manners (Harrington and Gould, 2015; Ru et al., 2023a). Different assumptions about the functional form of the temperature effects when the model was originally built resulted in diverse models. For more information and detailed descriptions of the above phenological model, please refer to Section 2.3.3 in the Supporting Materials.

**Table 3:** Temperature response functions and structures of chilling/forcing-based apple phenology models used in this study.

Models	Functions	Parameters	Description
M1	$\sum_{t_{1}}^{DOY}R_{\text{f}}\left(T_{\text{i}}\right)\geq {\left(L_{i}/24\right)}^{\text{k}}F^{*}$ (1) $\begin{array}{ll} R_{f}\left(T_{i}\right)= & \max\left(T_{i}-threshold,0\right) \end{array}$ (2)	t_1_ T F* k	The DOY which forcing accumulating beings The threshold above which forcing accumulates The total forcing units required Daylength coefficient
Uniforc	$\sum_{\text{t}=t_{1}}^{DOY}R_{f}(T_{\text{i}})\geq F^{*}$ (3) $R_{\text{f}}(T_{i})=\frac{1}{1+e^{b\left(T_{i}-c\right)}}$ (4)	t_1_ F* b c	The DOY which forcing accumulating beings The total forcing units required Sigmoid function parameter Sigmoid function parameter
Alternating	$\sum_{t_{1}}^{DOY}R_{f}(T_{i})\geq a+be^{c\left(t\right)}$ (5) $\begin{array}{ll} R_{f}\left(T_{i}\right)= & \max\left(T_{i}-threshold,0\right) \end{array}$ (6)	t_1_ threshold a b c	The DOY which forcing accumulation starts. Degree threshold above which forcing accumulates, and below which chilling accumulates. Intercept of chill day curve Slope of chill day curve scale parameter of chill day curve
Unichill	$\sum_{t_{1}}^{DOY}R_{f}(T_{\text{i}})\geq F^{*}$ (7) $\sum_{t_{0}}^{t_{c}}R_{c}(T_{j})\geq C^{*}$ (8) $R_{f}(T_{i})=\frac{1}{1+e^{b\_f\left(T_{i}-c\_f\right)}}$ (9) $\begin{array}{ll} R_{c}(T_{j})= & \frac{1}{1+e^{a\_c{\left(T_{i}-c\_c\right)}^{2}}{+}^{b\_c(T_{i}-c\_c)}} \end{array}$ (10)	t_0_ C* F* b_f c_f a_c b_c c_c	The DOY which chilling accumulating beings The total chilling units required The total forcing units required Sigmoid function parameter for forcing Sigmoid function parameter for forcing Sigmoid function parameter for chilling Sigmoid function parameter for chilling Sigmoid function parameter for chilling

These models are driven by daily response temperature and daylength after a starting date. R_f_ = daily sum of rates of forcing; R_c_ = daily sum of rates of chilling; T_i_ = the response temperature to forcing of the Julian day i; T_j_ = the response temperature to forcing of the Julian day j; DOY = Day of the year.

The core weighted model was an overall model that combined the predictions of several phenological models. The variance of the output value of this weighted model is lower and better than any member of the model. The weight (w_m_) of this core model is determined by the Root Mean Square Error (RMSE) values for each participating models (m) in model validation. If m*_RMSE_* is the RMSE in the phenological model validation of each participating model, $w_{\text{m}}=\frac{1}{m_{RMSE}\cdot \sum \frac{1}{{\text{m}}_{RMSE}}}$ . Therefore, the final simulated apple flowering dates were actually weighted ensembles of four different models.

#### 2.3.4 Model calibration and performance evaluation

The phenological model was subjected to rigorous calibration and validation procedures before application. Initially, the basin hopping algorithm was employed to estimate the parameter values of the phenology model (Wales and Doye, 1997). This calibration process minimized the RMSE (Equations 11) between each model’s simulated and observed apple phenology dates. To achieve the most accurate model parameters, the basin hopping algorithm was configured to run for 1000 iterations, accompanied by a random perturbation value of 0.5. The calibration process was iterated until parameters yielding the final selected minimum RMSE value were obtained (Wang et al., 2021b). Subsequently, the remaining 30% of phenological observations were used to evaluate the robustness of the apple phenology simulation model during the testing process. Additionally, we computed the overall adjusted R square (R^2^, Equations 12) to evaluate the goodness-of-fit and prediction error measures during both the calibration and validation datasets.


(11) $$RMSE=\sqrt{\frac{\sum_{i=1}^{n}{\left(O_{i}-P_{i}\right)}^{2}}{n}}$$

(12) $$R^{2}\frac{=1-\sum_{i}{\left(O_{i}-P_{i}\right)}^{2^{\;}}}{\sum_{i}{\left(O_{i}-{\bar{O}}_{i}\right)}^{2}}$$

where *O_i_* represented observed apple phenology dates (DOY); $\bar{O}$ represented the average observed phenology dates (DOY); *P_i_* represented simulated phenology dates at site *i*; *n* was the number of observations.

### 2.4 Quantifying apple water suitability level using the FAO 56 Penman-Monteith ET_o_ equation

#### 2.4.1 Estimating water requirements for apples

Apple water requirement (WR) refers to the total amount of water required to compensate for evapotranspiration losses from farmland under well-managed conditions, such as the absence of water, fertilizer, or insect stress (Doorenbos et al., 1977; Villalobos et al., 2016). The specific water requirement of apples is calculated by multiplying the specific coefficient parameters of apple crops at different growth stages and the reference evapotranspiration (Equation 13). The formula is as follows:


(13) $$WR_{j}=\sum_{\text{i}=1}^{n}K_{cj}\times ET_{oi}$$

Where WR is apple water requirement (mm); *j* is the key phenological stage of apple (flowering and fruit setting period, fruit enlargement period and coloring and maturity period); n is the number of days included in the j^th^ growth period of apple, and *i* is the day ordinal number. *K_c_* is the crop coefficient in the j^th^ growth period, which represents the comprehensive influence coefficient of various impedances (such as surface impedance, stomatal impedance, and diffusion impedance). ET_o_ is the reference crop evapotranspiration (mm/d) (Equation 14). The formula based on the FAO 56 Penman-Monteith (P-M) ET_o_ (Allen et al., 1998; Villalobos et al., 2016) estimation formula is as follows:


(14) $$ET_{\text{o}}=\frac{0.408\Delta (R_{n}-G)+\gamma \frac{900}{T+273}u_{2}(e_{s}-e_{a})}{\Delta +\gamma (1+0.34u_{2})}$$

Where *ET_o_* represents the reference crop evapotranspiration (mm/day); *R_n_* denotes the net radiation input within the canopy [MJ/(m^2^ day)]; G stands for the soil heat flux [MJ/(m^2^ day)]; *T* represents the daily average temperature at a high altitude of 2 meters (°C); *u2* signifies the wind speed at a height of 2 meters (m/s), derived from the wind speed value *u_10_* measured at 10.5 meters by the weather station, following the unified standard, where *u_2_* = 0.743 *u10*; *e_s_* represents the saturated water vapor pressure (kPa); *e_a_* represents the actual water vapor pressure (kPa); *Δ* denotes the slope of the curve representing the relationship between the difference in saturated water vapor pressure and temperature at a specific point (kPa/°C); *γ* symbolizes the constant relating to wet and dry thermometers (kPa/°C). For clarity, all the above parameters, parameter symbols, and calculation methods involved in the ET_o_ formula are presented in **Table 4**.

**Table 4:** Each parameter, parameter symbols, and calculation methods including in ET_o_ formula.

No.	Parameter	Symbol	Formula
1	Saturated vapor pressure	*e (T)*	$e(T)=0.6108\times \exp(\frac{17.27T}{T+237.3})$
2	Average saturated vapor pressure	*e_s_*	$e_{s}=\frac{e(T_{\max})+e(T_{\min})}{2}$
3	Actual water pressure	*e_a_*	$e_{a}=\frac{RH}{100}\times e_{s}$
4	The slope of the relationship curve between saturated water vapor pressure difference and temperature at a certain point	Δ	$\Delta =\frac{4098e_{a}}{{\left(T+237.3\right)}^{2}}$
5	Atmospheric pressure	*P*	$P=101.3\times {\left(\frac{293-0.0065z}{293}\right)}^{5.26}$
6	Latent heat of water	λ	$\lambda =2.501-(2.361\times 10^{-3})\times T$
7	Wet and dry thermometer constants	*γ*	$\gamma =0.00163\frac{P}{\lambda }$
8	Wind speed at 2 meters	*μ_2_*	$u_{2}=\frac{4.87\times u_{10}}{\log(678-5.42)}$
9	Average distance between sun and earth	*dr*	$dr=1+0.033\times \cos(\frac{2\pi }{365}J)$
10	Sun tilt angle	*δ*	$\delta \mathrm{=-0}.4093\times \text{cos(}\frac{2\pi J}{365})$
11	Angle of sunshine hours	*w_s_*	$w_{s}=\arccos(-\tan\varphi \tan\delta )$
12	Solar radiation at the top of the atmosphere	*R_a_*	$R_{a}=\frac{24\times 60\times 0.082}{\pi }\times dr\times (w_{s}\sin\varphi \sin\delta +\cos\varphi \cos\delta \sin w_{s})$
13	Maximum possible sunshine hours	*N*	$N=\frac{24}{\pi }w_{s}$
13	Maximum possible sunshine hours	*N*	$N=\frac{24}{\pi }w_{s}$
14	Net longwave radiation	*R_nl_*	$\begin{array}{l} R_{nl}=2.45\times 10^{-9}\times (0.1+\frac{0.9n}{N})\times (0.34-0.14\sqrt{e_{a}})\;\\ \times \;({\left(T_{\max}+273\right)}^{4}+{\left(T_{\min}+273\right)}^{4}) \end{array}$
15	Net shortwave radiation	*R_ns_*	$R_{ns}=0.77\times (0.25+0.5\frac{n}{N})\times R_{a}$

#### 2.4.2 Determining irrigation water requirements for apples

The apple irrigation water requirement (IWR) refers to the volume of water essential to satisfy the physiological water requirement (WR) of apple trees, in addition to the precipitation received (Equation 15). The calculation formula is outlined below:


(15) $$IWR_{j}=\{\begin{array}{c} WR_{j}-P_{eff,\;j},\;WR_{i}>P_{eff,\;j}\;\\ 0\;,\;WR_{j}\leq P_{eff,\;j} \end{array}$$

Where *IWR* represents the irrigation water requirement of apples (mm), *WR* denotes the water requirement of apples (mm), *P_eff_* signifies the effective precipitation (mm), and *j* corresponds to the critical phenological stage of apples (including flowering-fruit setting, fruit expansion, and coloring-maturity stage).

Given that not all precipitation (*P*) received in the field is available for crop utilization, this study incorporates the concept of effective precipitation (*P_eff_*). Effective precipitation refers to the portion of precipitation that actively contributes to soil conservation and plant growth under conditions conducive to meeting the water demands of plants. In the realms of irrigation and agriculture, understanding effective precipitation is pivotal for accurately computing irrigation water needs and judiciously managing water resources in agricultural land. *P_eff_* represents the fraction of total precipitation (*P*) that can directly benefit crops without loss. The variability in *P_eff_* is influenced by several factors, including precipitation characteristics, soil parameters, crop evapotranspiration rates, and irrigation practices. In this study, the United States Department of Agriculture (USDA) soil conservation method was employed (Bal et al., 2022), and *P_eff_* was estimated using the following formula (Equation 16). Detailed analytical data and results on *P_eff_* in the study area are provided in the Supporting Material (**Supplementary Figure S2**).


(16) $$P_{eff}=\{\begin{array}{c} \frac{P(4.17-0.2P)}{4.17}\;,P<8.2\;\text{mm}\\ 4.17+0.1P\;,\;P\;\geq 8.2\;\text{mm} \end{array}$$

#### 2.4.3 Assessing levels of water suitability for apples

The water suitability level (S) for apples is quantified by the extent to which the cumulative effective precipitation during the apple’s growth period fulfills its physiological water needs (Equation 17). This relationship is captured by the formula:


(17) $$S_{j}=\frac{P_{eff,\;j}}{WR_{j}}$$

Where *S_j_* represents the water suitability level of apples in the *j^th^* growth period, *P_eff_* represents the cumulative effective precipitation during apple corresponding phenological phases (mm), and *WR_j_* represents the physiological water demand of apples during the corresponding phenological phases (mm);

This study classified six water suitability levels for apples (Equation 18) according to the studies on water suitability level indicators (Oiu et al., 2018). Each level corresponds to a specific range of water suitability, indicating how well the apple trees’ water requirements are being met.


(18) $$\text{Level}(S_{j})=\{\begin{array}{c} \begin{array}{c} Excessive\;moisture,\;S_{j}\geq 2.0\\ High\;suitability,\;1.0\leq S_{j}<2.0\;\\ Good\;suitability,\;0.7\leq S_{j}<1.0 \end{array}\\ Suitability,\;0.5\leq S_{j}<0.7\\ Not\;suitability,\;0.4\leq S_{j}<0.5\\ Worst\;suitability,\;S_{j}<0.4 \end{array}$$

## 3 Results

### 3.1 Performance of models and phenology time derived from simulation

**Figure 3:**
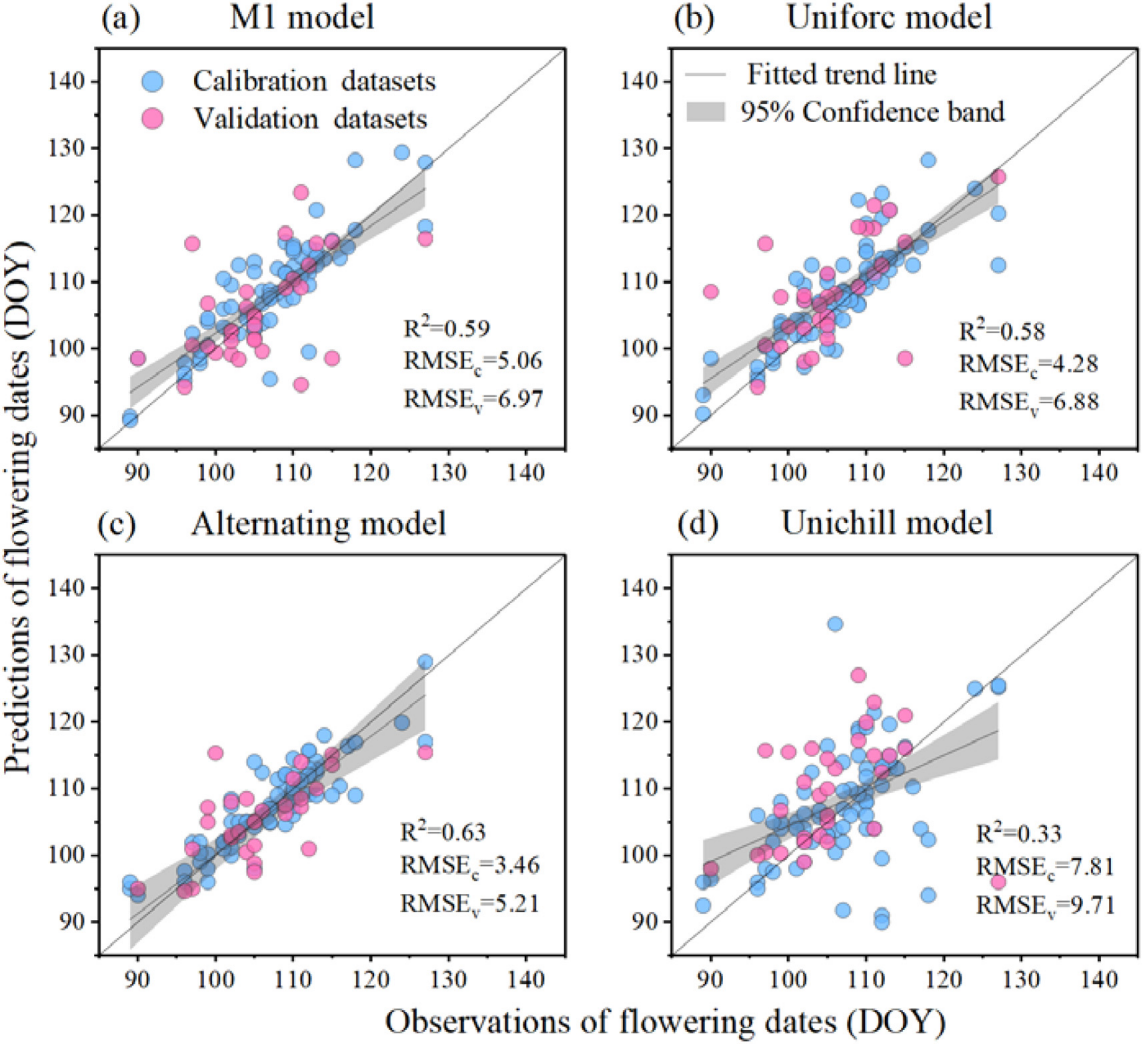
Root-mean-square error (RMSE) and R^2^ between apple flowering observations and predictions in model calibration (RMSE_c_) and validation (RMSE_v_). The blue circles represented the calibration dataset, the pink circles represented the validation dataset, the gray line indicated the linear trend line, and the gray shaded area denoted the 95% confidence band.

The performances of four phenological models in predicting apple initial flowering dates were evaluated using the R^2^ and RMSE values in the model validation process (or RMSE_v_), as depicted in **Figure 3**. Among the predicted apple flowering dates of the other three models, the Unichill model exhibited the largest simulation error (R^2^ = 0.33, RMSEv = 9.71 d), while the Alternating model demonstrated the best simulation accuracy (R^2^ = 0.63, RMSE_v_ = 5.21 d). The M1 model yielded a slightly larger RMSE_v_ value of 6.97 d (R^2^ = 0.59), and the Uniforc model had an RMSE_v_ value of 6.88 d (R_2_ = 0.58). Based on these values obtained from the model validation process, it can be concluded that the performances of the four phenological models in predicting apple flowering dates were generally acceptable for simulation studies.

**Figure 4:**
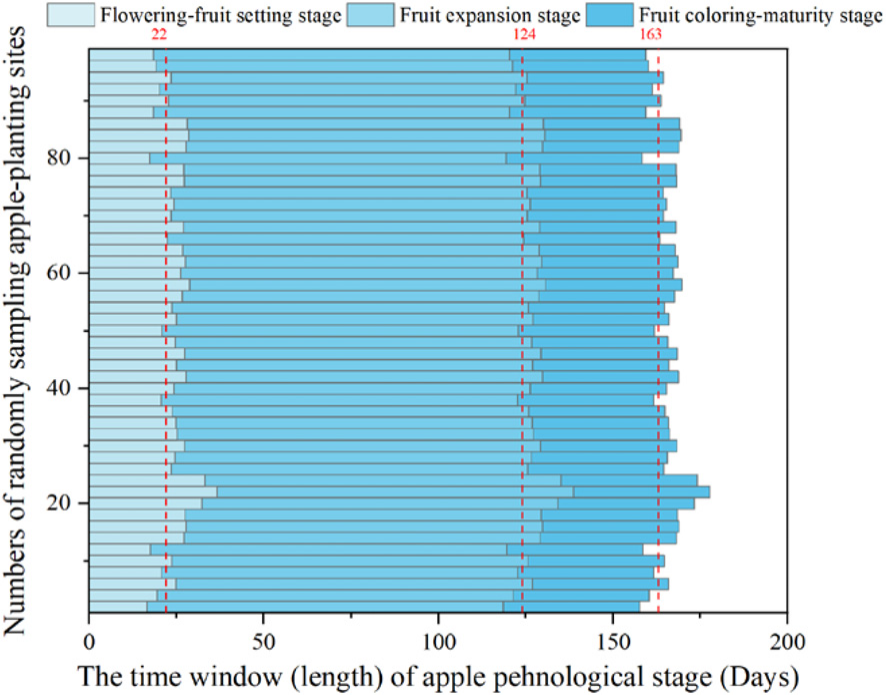
The time window for the apple flowering-fruit setting, fruit expansion, and fruit coloring-maturity stages. These phenological time was quantified based on the simulated apple flowering dates obtained from the calibrated phenological model mentioned above. The red dotted line is the average line.

After modeling the flowering dates of various regions, we computed the time window from initial flowering to fruit set, initial fruit expansion to final fruit expansion, and fruit coloring to maturity stages, respectively (**Figure 4**). It is important to note that our study encompassed over 300 apple-producing counties across China. To present the quantitative findings effectively, we randomly selected the results from 100 sites for display. The average length of apple flowering-fruit setting, fruit expansion, and fruit coloring-maturity stages was 22, 102, and 39 days, respectively. The three phenological stages of apples differ across different regions, which displayed the advantage of individually calculating different phenological stages of apples based on modeling methods. This approach highlighted the importance of using phenological models to simulate the flowering dates when considering the external climate conditions.

### 3.2 Spatiotemporal changing trends of apple water requirements

**Figure 5:**
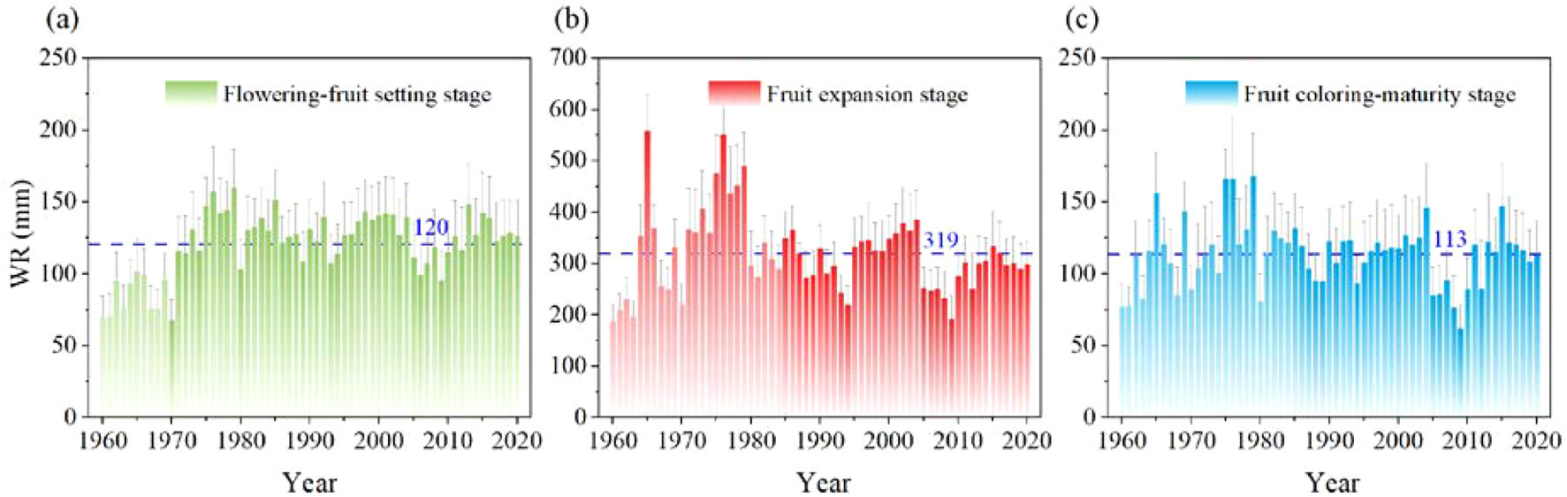
Temporal variation of water requirement (WR) in apple flowering-fruit setting stage **(a)**, fruit expansion stage **(b)**, and fruit coloring-maturity stage **(c)** from 1960 to 2020 in the whole apple-producing areas of China.

Assessing the spatial and temporal variation in water requirements for apples is crucial for effective water management and sustainable apple production practices. The multi-year average water requirements (WR) of apples were 120 mm, 319 mm, and 113 mm during the flowering-fruit setting stage, fruit expansion stage, and coloring-maturity stage, respectively (**Figure 5**). The results showed that the WR was highest during the fruit expansion stage, followed by the flowering-fruit setting stage and the coloring-maturity stage. This trend suggested that more water was needed during the stages of active growth and fruit development, which aligned with the physiological demands of the apple tree. The minimum and maximum WR were approximately 67 mm and 160 mm during the flowering-fruit setting stage, respectively. During the fruit expansion stage, the minimum and maximum WR were approximately 191 mm and 559 mm, respectively, and approximately 62 mm and 167 mm during apple coloring-maturation stage. The variability in the range of WR values for each stage indicated the varying water needs within each apple phenological stage, which highlighted the importance of effectively understanding and managing water consumption throughout the apple growing season.

**Figure 6:**
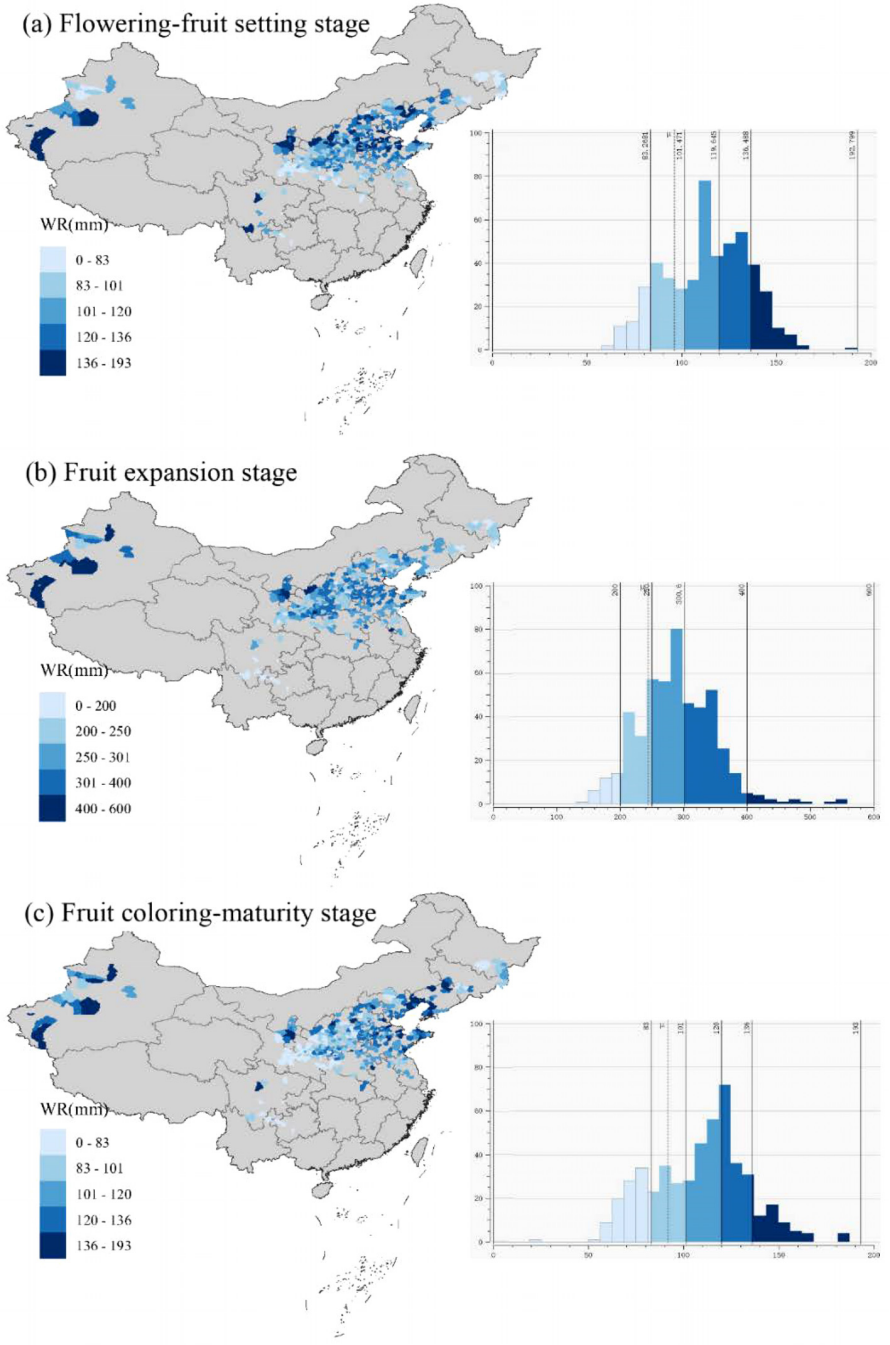
Spatial distribution map and frequency histogram of water requirement (WR) during apple flowering to fruit-setting stage **(a)**, fruit expansion stage **(b)**, and fruit coloring maturity stage **(c)** in the whole apple-producing areas of China.

The changes of WR were analyzed through the spatial distribution map and the frequency distribution histogram during apple flowering-fruit-setting, fruit expansion and coloring-maturity stage (**Figure 6**). The average WR of apples were 114 mm, 290 mm, and 109 mm during the flowering-fruit setting stage, fruit expansion stage, and coloring-maturity stage across all apple production counties, respectively. Some apple production areas exhibited relatively high water requirements (WR > 136 mm) during the flowering-fruit setting stage. These areas included the northwestern parts of the Loess Plateau (e.g., Ningxia, Shaanxi, and northern Shanxi province), the central Bohai Bay areas, and the southern parts of Xinjiang. Conversely, areas with relatively low water requirements (WR< 83 mm) were found in eastern parts of Gansu, Henan, and Heilongjiang province during apples flowering-fruit setting stage. The frequency diagram displayed the normal distribution trend of apple WR during the three phenological stages. The WR of 101 to 120 mm was at the maximum frequency during the flowering-fruit setting stage (**Figure 6a**). During the fruit expansion stage, the WR of the apple notably surpassed that of the flowering-fruit setting stage. Areas with higher water requirements were identified in Xinjiang, Ningxia, Shaanxi, and Shanxi provinces (**Figure 6b**). The WR in the range of 250 to 301 mm was at the maximum frequency during apple fruit expansion stage. In contrast, the WR was slightly lower during the coloring-maturation stage, with the range of 101 to 136 mm of WR showing the maximum frequency compared to the fruit expansion stage (**Figure 6c**).

### 3.3 Spatiotemporal changing trends of apple irrigation water requirements

**Figure 7:**
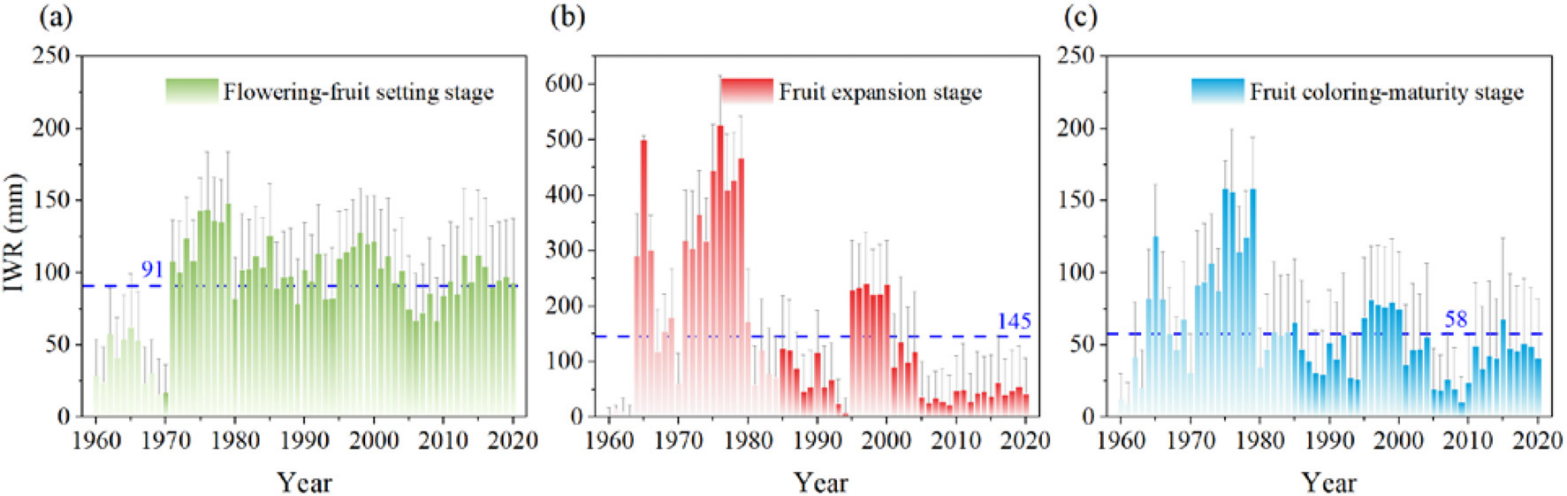
Temporal variation of irrigation water requirement (IWR) in apple flowering-fruit setting stage **(a)**, fruit expansion stage **(b)**, and fruit coloring-maturity stage **(c)** from 1960 to 2020 in the whole apple-producing areas of China (The blue dotted line is the average IWR line).

The irrigation water requirements (IWR) were accessed during three phenological stages across apple-producing counties in China (**Figure 7**). The average IWR of apples can be ranked as follows: fruit expansion stage (145 mm) > flowering-fruit setting stage (91 mm) > coloring-maturity stage (58 mm). The IWR during the flowering–fruit setting stage ranged from 16 mm to 148 mm, with a standard deviation of approximately ±34 mm. This indicated moderate interannual variability and suggested that a fixed irrigation amount could generally meet the needs during this stage. In contrast, the IWR during the fruit expansion stage showed the greatest variability, ranging from 4 mm to 524 mm, with a standard deviation of ±41mm. This reflected a high sensitivity to climatic fluctuations and emphasized the importance of dynamic irrigation scheduling. During the coloring–maturity stage, the IWR ranged from 3 mm to 196 mm, with relatively lower overall water demand but still observable interannual variation.

**Figure 8:**
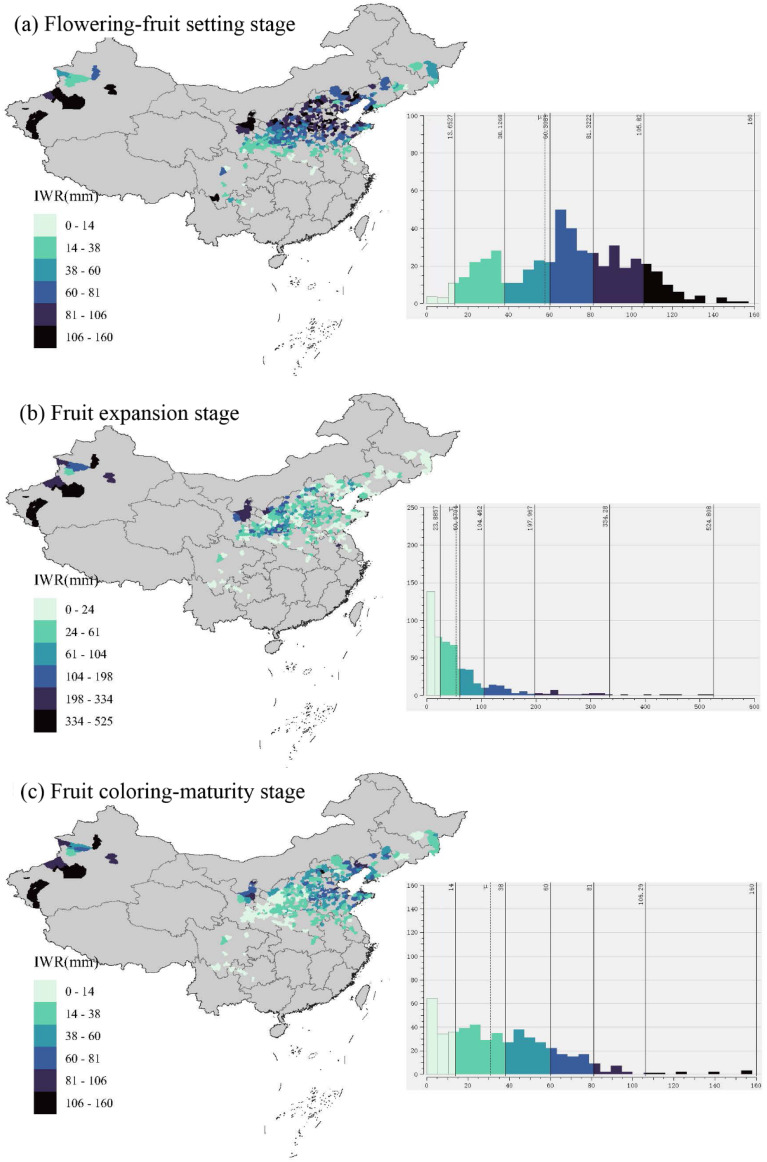
Spatial distribution map and frequency histogram of irrigation water requirement (IWR) during apple flowering-fruit setting stage **(a)**, fruit expansion stage **(b)**, and fruit coloring-maturity stage **(c)** in the whole apple-producing areas of China.

The spatial distribution map and the frequency distribution histogram of apples’ IWR were shown during three phenological stages (**Figure 8**). As latitude increases, the IWR also increases during the flowering-fruit-setting stage. In the northern high latitudes (e.g., Xinjiang production areas), the IWR was the highest. This phenomenon could be attributed to China’s precipitation pattern, where it rains more in the southeast and less in the northwest. Regionally, the Bohai Bay apple production area exhibited high average IWR during the apple flowering-fruit setting stages, while Xinjiang and the Loess Plateau (e.g., Gansu, Ningxia, and Shaanxi) production areas showed high IWR during the fruit expansion stages and the coloring-maturation stage. Additionally, the frequency histogram revealed that the IWR was most concentrated between 60 and 81 mm during the flowering-fruit setting in apple-producing counties across the country, 0 and 24 mm during the fruit expansion stage, and 14 and 38 mm during the coloring-maturity stage.

### 3.4 Spatiotemporal changing trends of apple water suitability level

**Figure 9:**
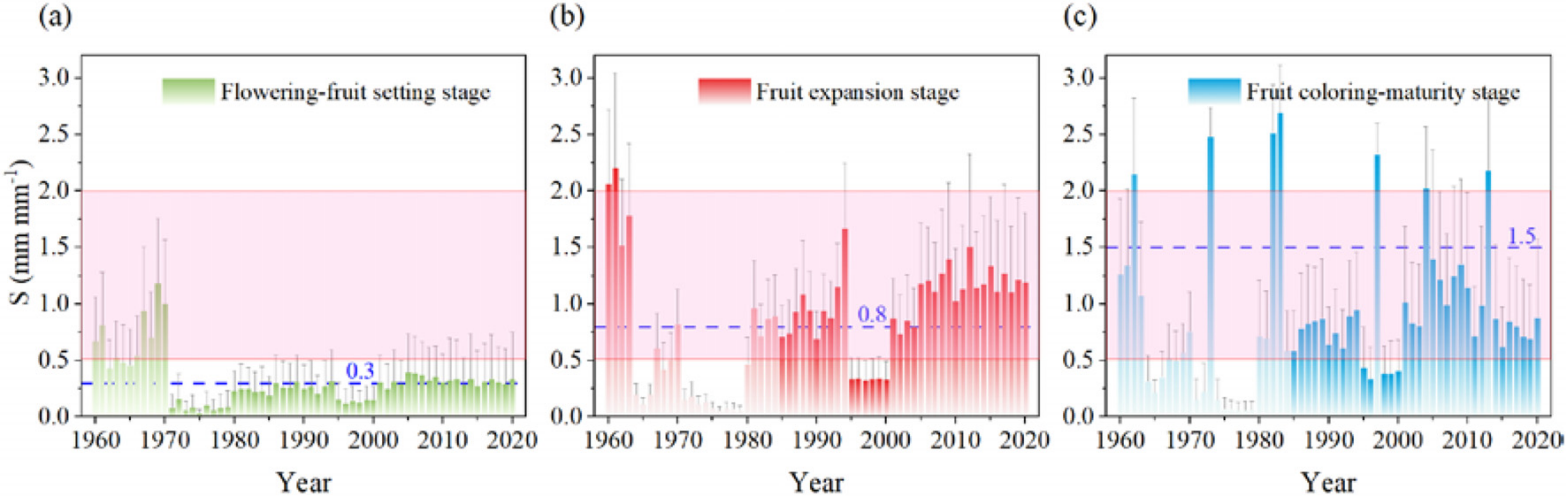
Temporal variation of water suitability (S) in apple flowering-fruit setting stage **(a)**, fruit expansion stage **(b)**, and fruit coloring-maturity stage **(c)** from 1960 to 2020 in the whole apple-producing areas of China (The blue dotted line denotes the average line, while the light pink area represents the range from the suitable level to the extremely suitable level of apple water requirements.).

The level of water suitability (S) of apples was assessed over time and space in apple production areas of China based on the concepts of effective precipitation and apple water requirements (**Figure 9**). The annual average water suitability of apples was 0.3 (unsuitable), 0.8 (fairly suitable), and 1.5 (extremely suitable) during the flowering-fruit setting stage, fruit expansion stage, and coloring-maturity stage, respectively. Specifically, during the flowering-fruit setting stage, the water suitability for apples reached an extremely suitable level in 1968, with a level of 1.18. However, from 1970 to 2020, the water suitability of apples during this stage remained consistently low. These findings underscored the prevalence of water scarcity during the apple flowering and fruit-setting stage in China over numerous years. During the fruit expansion stage, the highest water suitability occurred in 1962, with the highest value of 2.19, which was an excessive moisture level. We also found the water suitability was in the suitable and extremely suitable range (light pink area in **Figure 9**) during this stage of apples in most years. Moreover, the inter-annual fluctuations of water suitability exhibited considerable variability during apples during the coloring-maturity stage. These analyses showed more attention should be paid to the water suitability during the flowering-fruit setting stage.

**Figure 10:**
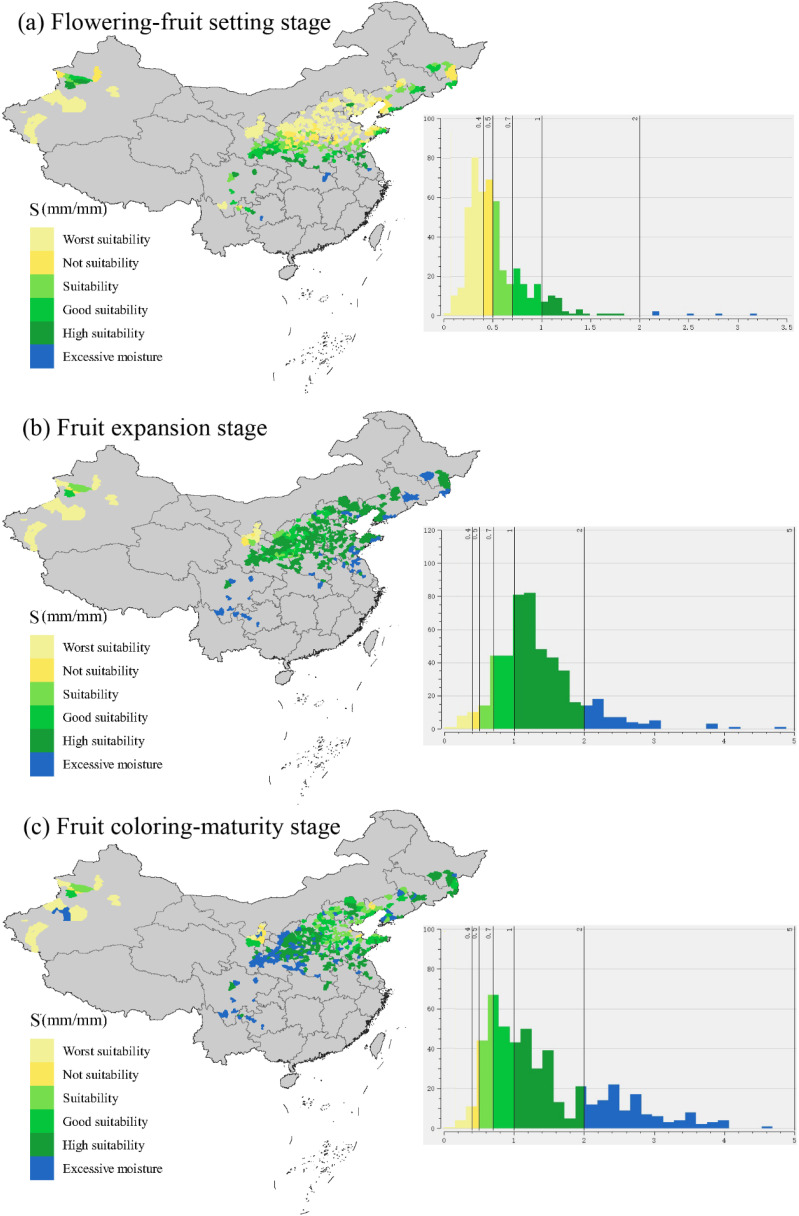
Spatial distribution map and frequency histogram of water suitability (S) during apple flowering-fruit setting stage **(a)**, fruit expansion stage **(b)**, and fruit coloring-maturity stage **(c)** in the whole apple-producing areas of China.

The spatial distribution map and histogram showed that the water suitability of most apple-producing counties in China was less than 0.4 (**Figure 10a**) during the flowering-fruit-setting stages, which was an extremely unsuitable level. These areas were mainly concentrated in Xinjiang apple production areas, the northwest parts of Loess Plateau production areas and the northern parts of Bohai Bay production areas. Conversely, during the fruit expansion stage, the water suitability level in most areas was extremely suitable, ranging from 1.0 to 2.0 (**Figure 10b**). The apple production areas of the Southwestern Cool Highlands exhibited excessively high water suitability levels (>2.0), and the few areas (e.g. Xinjiang and Ningxia province) experienced water shortages. During the apple coloring-maturation stage, the areas with excessive humidity not only included the Southwestern Cool Highlands but also extended to some counties in the southwestern Loess Plateau (**Figure 10c**).

## 4 Discussion

### 4.1 Enhanced precision in apple water requirement estimation through dynamic phenological modeling

This study assessed apple water requirements by integrating accurate phenological simulation. The phenology modeling analysis showed that the Alternating model exhibited the highest accuracy, while the Unichill model showed the largest simulation error. The model ensemble method addressed the issue of low accuracy caused by arbitrary model selection (Dai et al., 2022; Ru et al., 2023b). Then, the time window of the flowering-fruit setting, fruit expansion, and fruit coloring-maturity stages was quantified. Previous studies used the Penman-Monteith equation to assess crop water requirements, it was common practice to vaguely define flowering and fruit-setting stages of all study areas within a broad time range, such as considering the period from March 1 to April 30 as the flowering and fruit-setting stage (Liu et al., 2024; Nyolei et al., 2019). In contrast, our study offered a more targeted and location-specific phenology dates (**Figure 4**). The dynamic phenological data simulated by the phenological model enabled the calculated apple irrigation water requirement to be adjusted according to the length of the phenological dates. For instance, when the temperature is high in a certain year, flower-fruiting is likely to occur earlier, and the water requirement may also be earlier. The data simulated by the phenological model could dynamically match the temporal distribution of apple water requirements.

### 4.2 Water requirement dynamics in different phenophases and regions

An in-depth analysis of the details of water requirements throughout the various stages of apple growth formed the foundation of sustainable apple cultivation practices. According to our results, the stages of apple flowering-fruit setting, fruit expansion, and coloring-maturity showed distinctly different water requirements (**Table 5**). At the beginning of the flowering-fruit setting stage, the apple tree began its reproductive process, a modest water requirement of about 120 mm was essential for maintaining the delicate water balance in apples. This finding is corroborated by the conclusions of a field-scale fruit study conducted in the United States (Dombrowski et al., 2022). As the apple tree progressed into the fruit expansion stage, characterized by vigorous growth and cellular proliferation, the water requirement increased dramatically. With an average requirement of 319 mm, this stage became the peak of water consumption, reflecting the heightened metabolic activity involved in fruit development (Moriondo et al., 2015). Subsequently, as the apple approached the coloring-maturity stage, there was a shift from vegetative to reproductive functions, accompanied by a corresponding decrease in water requirements. Despite this reduction, an average demand of 113 mm persisted, highlighting the continued importance of water management throughout the growing season. Importantly, the observed trend of increasing water requirements during stages of active growth and development aligned well with the physiological demands of the apple tree as previously reported (Dang et al., 2020; Wang et al., 2021a).

**Table 5:** Observed Water Requirements (WR) of 3 representative apple producing counties (Yuncheng [YC], Luochuan [LC], and Beijing [BJ]) and the indicator of WR, Irrigation Water Requirements (IWR), and Suitability (S) estimated by FAO 56 Penman-Monteith (P-M) ETo equation.

Phenophical stages	Observed WR (mm)			WR (mm)	IWR (mm)	Suitable (mm/mm)
	YC	YC	LC			
Flowering-fruit setting stage	112	94	132	120 ± 22	91 ± 34	0.31
Fruit expansion stage	320	274	340	319 ± 54	144 ± 41	0.91
Fruit coloring-maturity stage	102	88	105	113 ± 24	58 ± 12	1.50

The estimated WR, IWR, and S values represent averages derived from multi-year (1960–2020) data across 367 major apple-producing counties.

The combination of spatial analysis and frequency distribution histograms provided additional insights from an inter-regional perspective on dynamic water requirements. During the flowering-fruit setting stage, regions such as the northwestern areas of the Loess Plateau and the central Bohai Bay areas emerged as areas with elevated water needs. Conversely, there were areas with diminished water requirements along the eastern edges of Gansu, Henan, and Heilongjiang provinces. The accompanying frequency distribution histogram revealed the peak frequency of the normal distribution within the 101 to 120 mm range of water requirement. These statistical findings not only confirmed the spatial differences observed but also reinforced the prevalence of moderate water requirements during this stage of apple development. Transitioning into the fruit expansion stage, regions spanning Xinjiang, Ningxia, Shaanxi, and Shanxi provinces emerged as focal points of heightened water requirements. The frequency distribution histogram echoed this narrative, showing a notable shift towards higher ranges of water requirement, with the 250 to 301 mm range being the central point of frequency distribution. As the apple tree progressed towards the coloring-maturity stage, there was a subtle adjustment in water requirements, reflecting the transition from vegetative to reproductive functions (Juillion et al., 2022; Ye et al., 2020). The frequency distribution histogram demonstrated this trend, with the 101 to 136 mm range of water requirement dominating the frequency distribution landscape, representing the nuanced adjustments in water needs during apple maturation.

### 4.3 Variations of irrigation water requirements in different phenophases and regions

For irrigation water requirements (IWR) of apples, the order was as follows: fruit expansion > flowering-fruit setting > coloring-maturity. The differential IWR inherent in each phenophical phase aligned with the WR pattern of apples. The large difference between the average stability and interannual variation (standard deviation) of IWR at each stage reflected the dynamic impact of climatic factors on apple irrigation water requirements in the three phenological stages of apple (Gao et al., 2021; Gush et al., 2019). Spatially, the results unveiled a compelling correlation between latitude and IWR, with higher latitudes corresponding to elevated water demands during the flowering-fruit setting stage (Gao et al., 2018). This spatial gradient mirrored China’s precipitation patterns, with regions such as Xinjiang exhibiting the highest IWR due to diminished rainfall. Regionally, the Bohai Bay and Loess Plateau regions emerged as focal points of heightened water demand during distinct phenological stages, indicative of localized climatic influences shaping orchard water requirements. Complementing our spatial analysis, frequency distribution histograms showed the prevalence of specific IWR ranges across apple-producing counties. Notably, the concentration of IWR between 60 and 81 mm during the flowering-fruit setting stage underscored the prevalence of moderate water demands during this critical phase of apple development. In essence, the amalgamation of spatial analysis and temporal variability offered a comprehensive understanding of the complex nexus between climatic factors, geographical gradients, and agronomic demands shaping irrigation water requirements within apple-producing regions of China. However, this study did not perform separate analyses of specific biotic and abiotic stressors—such as diseases, pests, soil salinization, or nutrient deficiencies—which are known to significantly influence apple water use efficiency and suitability. Future research could benefit from incorporating crop–environment–stress coupling models, such as integrating disease indices into water suitability assessments or employing crop growth models to simulate water demand dynamics under varying stress conditions.

### 4.4 Water suitability dynamics in different phenophases and regions

The assessment of water suitability (S) in apple production counties of China offered valuable insights into the dynamic interplay between effective precipitation, apple water requirements, and regional climatic conditions. During the flowering-fruit setting stage, while a notable exception occurred in 1968, when water suitability reached an extremely suitable level, subsequent years witnessed consistently low water suitability levels, indicating recurrent water stress during this pivotal phase of apple development. During the fruit expansion stage, a contrasting picture emerged, characterized by a predominance of suitable to extremely suitable water suitability levels in most years. However, a notable anomaly in 1962, when excessively high water suitability levels were observed, highlighted the potential risks associated with excessive moisture during this developmental phase. Despite this anomaly, the overall trend underscored the favorable water conditions for fruit growth, development during the fruit expansion stage. The coloring-maturity stage unveiled a mosaic of spatial variability in water suitability, with regions experiencing both excessive humidity and water shortages. While areas such as the Southwestern Cool Highlands exhibited excessively high water suitability levels, extending beyond the optimal range, counties in the southwestern Loess Plateau faced challenges associated with excessive humidity.

Spatial analysis further clarified the disparities in water suitability across apple-producing counties, with concentrations of extremely unsuitable levels during the flowering-fruit setting stage in Xinjiang, the northwest parts of the Loess Plateau, and the northern parts of the Bohai Bay production areas. Conversely, during the fruit expansion stage, most regions exhibited favorable water suitability levels, with exceptions noted in Xinjiang and Ningxia province, indicating localized water scarcity challenges. The coloring-maturation stage presented a nuanced spatial distribution, with areas of excessive humidity extending from the Southwestern Cool Highlands to select counties in the southwestern Loess Plateau. These spatial nuances showed the localized climatic influences shaping water availability during apple coloring-maturation stages. This emphasized the need for specific irrigation management plans to address risks related to water suitability challenges. Compared to previous research (Wang et al., 2020b; Ye et al., 2020), this study offers a more detailed understanding of spatial and temporal dynamics, enabling stakeholders to implement targeted interventions for improved water management practices.

## 5 Conclusion

This study provides a comprehensive macroscopic evaluation of apple water requirements and suitability levels at the county scale in China by integrating phenological modeling-derived phenophases with the FAO 56 Penman-Monteith (P-M) ET_o_ approach. The results reveal significant variation in water needs and suitability across different phenological stages and regions, highlighting the critical importance of tailored water management strategies. The highest average water requirements were observed during the fruit expansion stage, while the flowering-fruit setting stage exhibited the lowest water suitability, particularly in regions such as Xinjiang, the northwest Loess Plateau, and northern Bohai Bay. Conversely, excessive moisture was noted in the Southwestern Cool Highlands during the fruit expansion and coloring-maturity stages. These findings underscore the necessity for region-specific water management practices to enhance apple quality and sustainability. By identifying phenological windows with varying water suitability, this study offers valuable insights for optimizing irrigation practices and supports the sustainable development of the apple industry in China.

## Data Availability

The original contributions presented in the study are included in the article/**Supplementary Material**. Further inquiries can be directed to the corresponding author/s.
