# Supplementary material for: Assessing water requirements and suitability for apple growth at county scale in China: a phenological modeling approach during key growth stages
Source: Front Plant Sci. 2025 May 12;16:1572647. doi: 10.3389/fpls.2025.1572647 (PMC12104271; doi:10.3389/fpls.2025.1572647)
Supplement: Supplementary file 1 [file DataSheet1.docx]

**Supplemental materials**

Table S1. Basic information about the 80 meteorological stations with solar radiation observations in China. The precipitation is the annual mean value, while the other meteorological factors are daily mean values.

| No. | Station | Latitude | Longitude | Rs | n | Tmax | Tmean | Tmin | RH | P | U | Record period |
| --- | --- | --- | --- | --- | --- | --- | --- | --- | --- | --- | --- | --- |
|  |  | (N) | (E) | (MJm^-2^d^-1^) | (h) | (℃) | (℃) | (℃) | (%) | (mm y^-1^) | (ms^-1^) |  |
| 1 | Golmud | 36.4 | 94.9 | 19.1 | 8.4 | 13.0 | 5.3 | -14.0 | 32.4 | 43.0 | 2.6 | 1957-2017 |
| 2 | Xining | 36.7 | 101.8 | 15.9 | 7.3 | 14.0 | 6.0 | 0.0 | 56.2 | 385.5 | 15.0 | 1959-2017 |
| 3 | Naqu | 31.5 | 92.1 | 17.6 | 76.0 | 7.1 | -0.9 | -7.6 | 51.9 | 444.0 | 2.6 | 1961-2017 |
| 4 | Yushu | 33.0 | 97.0 | 16.6 | 6.8 | 12.0 | 3.5 | -3.0 | 53.7 | 486.3 | 11.0 | 1960-2017 |
| 5 | Changdu | 31.2 | 97.2 | 16.8 | 65.0 | 168.0 | 7.8 | 1.0 | 50.3 | 477.6 | 11.0 | 1957-2017 |
| 6 | Ganzi | 31.6 | 100.0 | 18.1 | 6.9 | 14.8 | 6.3 | 0.2 | 55.8 | 659.1 | 1.8 | 1994-2017 |
| 7 | Hongyuan | 32.8 | 102.6 | 16.8 | 6.4 | 108.0 | 2.1 | -4.6 | 69.4 | 729.7 | 2.3 | 1994-2017 |
| 8 | Emeishan | 29.5 | 103.3 | 12.7 | 3.9 | 7.7 | 3.3 | 0.5 | 85.7 | 1763.2 | 3.0 | 1959-2017 |
| 9 | Leshan | 29.6 | 103.8 | 9.5 | 2.9 | 20.9 | 17.1 | 14.3 | 80.5 | 1323.1 | 1.3 | 1973-1990 |
| 10 | Zhaotong | 27.4 | 103.7 | 14.3 | 5.2 | 18.2 | 11.6 | 7.4 | 746.0 | 723.5 | 25.0 | 1961-1990 |
| 11 | Lijiang | 26.9 | 100.2 | 17.0 | 6.7 | 19.5 | 12.9 | 8.0 | 62.5 | 964.0 | 3.1 | 1961-2017 |
| 12 | Panzhihua | 26.6 | 101.7 | 16.1 | 7.4 | 27.8 | 20.9 | 15.7 | 56.9 | 816.4 | 1.4 | 1992-2017 |
| 13 | Weining | 26.9 | 104.3 | 13.1 | 49.0 | 16.3 | 10.4 | 6.8 | 79.8 | 927.0 | 3.2 | 1961-1990 |
| 14 | Tengchong | 25.0 | 98.5 | 15.2 | 5.9 | 21.6 | 15.2 | 10.6 | 77.4 | 1481.0 | 1.6 | 1957-2017 |
| 15 | Kunming | 25.0 | 102.7 | 15.1 | 6.2 | 21.1 | 15.2 | 10.6 | 71.4 | 987.1 | 2.1 | 1959-2017 |
| 16 | Ankang | 32.7 | 109.0 | 11.4 | 46.0 | 21.4 | 16.0 | 12.2 | 73.8 | 802.5 | 1.4 | 1990-2017 |
| 17 | Yichang | 30.7 | 111.4 | 10.9 | 4.2 | 21.6 | 17.0 | 13.6 | 75.3 | 1144.8 | 1.3 | 1957-2017 |
| 18 | Wuhan | 30.6 | 114.1 | 12.3 | 5.3 | 21.4 | 16.8 | 13.2 | 76.9 | 1262.4 | 2.0 | 1957-2017 |
| 19 | Shapingba | 29.6 | 106.5 | 8.6 | 2.7 | 22.4 | 18.6 | 15.9 | 78.4 | 1103.5 | 1.4 | 1987-2017 |
| 20 | Jishou | 28.2 | 109.7 | 9.6 | 3.4 | 21.8 | 17.0 | 13.8 | 79.1 | 1404.6 | 1.2 | 1992-2017 |
| 21 | Changsha | 28.1 | 112.8 | 10.7 | 4.2 | 21.8 | 17.6 | 14.6 | 77.9 | 1458.7 | 2.2 | 1987-2017 |
| 22 | Guiyang | 26.6 | 106.7 | 10.3 | 3.2 | 19.6 | 15.1 | 12.1 | 77.4 | 1091.7 | 2.3 | 1959-2017 |
| 23 | Changning | 26.4 | 112.4 | 11.0 | 3.7 | 22.8 | 18.5 | 15.4 | 77.4 | 1421.3 | 1.9 | 1992-2017 |
| 24 | Guilin | 25.3 | 110.3 | 11.5 | 41.0 | 23.4 | 19.0 | 16.0 | 74.9 | 1870.8 | 2.4 | 1957-2017 |
| 25 | Nanjing | 31.9 | 118.9 | 12.6 | 5.5 | 20.5 | 15.7 | 11.9 | 75.1 | 1074.0 | 2.5 | 1959-2017 |
| 26 | Lvsi | 32.1 | 121.6 | 13.2 | 6.0 | 19.4 | 15.8 | 12.9 | 78.2 | 1100.7 | 3.4 | 1992-2017 |
| 27 | Hefei | 31.8 | 117.3 | 12.3 | 5.3 | 20.6 | 16.1 | 12.4 | 75.3 | 1004.6 | 2.6 | 1959-2017 |
| 28 | Hangzhou | 30.2 | 120.2 | 11.8 | 48.0 | 21.2 | 16.8 | 13.4 | 76.5 | 1414.9 | 2.2 | 1959-2017 |
| 29 | Cixi | 30.2 | 121.3 | 12.7 | 5.6 | 20.4 | 16.2 | 12.9 | 81.0 | 1259.0 | 2.8 | 1961-1990 |
| 30 | Lushan | 29.6 | 116.0 | 13.2 | 5.0 | 15.3 | 11.6 | 8.8 | 78.0 | 1953.3 | 5.0 | 1960-1990 |
| 31 | Tunxi | 29.7 | 118.3 | 12.1 | 46.0 | 22.4 | 17.0 | 13.1 | 78.1 | 1806.5 | 1.3 | 1992-2017 |
| 32 | Nanchang | 28.6 | 115.9 | 12.4 | 5.1 | 21.8 | 17.9 | 14.9 | 76.0 | 1585.9 | 25.0 | 1959-2017 |
| 33 | Hongijia | 28.6 | 121.4 | 12.7 | 46.0 | 22.2 | 18.0 | 15.0 | 77.3 | 1590.7 | 2.3 | 1992-2017 |
| 34 | Jian'ou | 27.1 | 118.3 | 13.5 | 4.6 | 25.0 | 19.2 | 15.4 | 80.1 | 1742.2 | 1.4 | 1992-2017 |
| 35 | Fuzhou | 26.1 | 119.3 | 12.2 | 45.0 | 24.6 | 20.0 | 17.0 | 75.3 | 1389.5 | 2.6 | 1959-2017 |
| 36 | Shaoguan | 24.7 | 113.6 | 12.3 | 4.9 | 25.4 | 20.5 | 17.0 | 75.7 | 1500.0 | 1.4 | 1960-1990 |
| 37 | Guangzhou | 23.2 | 113.5 | 11.9 | 46.0 | 26.5 | 22.1 | 19.0 | 77.0 | 1781.5 | 1.9 | 1957-2017 |
| 38 | Shantou | 23.4 | 116.7 | 14.0 | 5.6 | 25.5 | 21.8 | 19.0 | 79.6 | 1568.4 | 25.0 | 1957-2017 |
| 39 | Nanning | 22.6 | 108.2 | 12.6 | 44.0 | 26.4 | 21.7 | 18.5 | 79.1 | 1297.3 | 15.0 | 1961-2017 |
| 40 | Zhongshan | 22.5 | 113.4 | 12.1 | 4.9 | 25.7 | 21.8 | 18.9 | 82.9 | 1801.8 | 2.1 | 1965-1990 |
| 41 | Beihai | 21.5 | 109.1 | 14.2 | 5.1 | 26.8 | 23.1 | 20.4 | 79.8 | 1828.2 | 3.3 | 1993-2017 |
| 42 | Mohe | 53.0 | 122.5 | 12.2 | 66.0 | 48.0 | -4.2 | -12.1 | 68.6 | 443.3 | 1.8 | 1993-2017 |
| 43 | Fuyu | 47.8 | 124.5 | 14.1 | 70.0 | 9.2 | 3.4 | -19.0 | 62.9 | 433.9 | 3.1 | 1993-2017 |
| 44 | Jiamusi | 46.8 | 130.3 | 12.4 | 66.0 | 9.4 | 3.5 | -2.1 | 66.5 | 534.4 | 3.1 | 1961-2017 |
| 45 | Harbin | 45.9 | 126.6 | 12.9 | 6.7 | 10.1 | 44.0 | -1.0 | 65.1 | 516.3 | 3.3 | 1959-2017 |
| 46 | Yuzhong | 35.9 | 104.2 | 15.3 | 7.0 | 14.6 | 7.4 | 1.6 | 62.1 | 359.0 | 2.1 | 2005-2017 |
| 47 | Datong | 40.1 | 113.4 | 15.4 | 7.4 | 14.1 | 7.0 | 0.8 | 52.3 | 374.6 | 2.8 | 1960-2017 |
| 48 | Taiyuan | 37.6 | 112.6 | 14.5 | 6.9 | 17.1 | 10.2 | 4.2 | 58.4 | 442.9 | 2.1 | 1959-2017 |
| 49 | Guyuan | 36.0 | 106.3 | 15.2 | 7.1 | 13.7 | 7.3 | 1.9 | 60.5 | 432.9 | 2.6 | 1985-2017 |
| 50 | Houma | 35.7 | 111.4 | 13.5 | 6.1 | 19.6 | 12.9 | 7.3 | 64.5 | 506.0 | 1.9 | 1959-2017 |
| 51 | Tongliao | 43.6 | 122.3 | 14.0 | 8.2 | 13.1 | 6.8 | 11.0 | 54.3 | 364.5 | 3.6 | 1960-2017 |
| 52 | Changchun | 43.9 | 125.2 | 13.6 | 7.1 | 11.3 | 5.7 | 0.7 | 62.9 | 584.6 | 3.7 | 1959-2017 |
| 53 | Yanji | 42.9 | 129.5 | 13.0 | 6.3 | 12.1 | 5.4 | -0.2 | 64.6 | 523.5 | 2.6 | 1960-2017 |
| 54 | Chaoyang | 41.6 | 120.4 | 14.2 | 74.0 | 16.1 | 9.2 | 2.9 | 51.7 | 467.7 | 2.8 | 1963-2017 |
| 55 | Shenyang | 41.7 | 123.5 | 13.5 | 68.0 | 14.1 | 8.3 | 3.1 | 63.5 | 688.7 | 2.9 | 1957-2017 |
| 56 | Beijing | 39.8 | 116.5 | 14.4 | 7.2 | 18.1 | 12.5 | 7.4 | 56.1 | 569.0 | 2.4 | 1957-2017 |
| 57 | Tianjin | 39.1 | 117.1 | 14.0 | 68.0 | 18.1 | 12.7 | 8.3 | 61.2 | 531.4 | 2.6 | 1959-2017 |
| 58 | Leting | 39.4 | 118.9 | 14.0 | 6.7 | 16.9 | 11.5 | 7.2 | 63.9 | 539.7 | 2.3 | 1992-2017 |
| 59 | Dalian | 38.9 | 121.6 | 13.7 | 7.4 | 14.7 | 11.1 | 8.1 | 64.7 | 615.5 | 4.4 | 1963-2017 |
| 60 | Jinan | 36.6 | 117.0 | 13.5 | 6.8 | 19.6 | 14.7 | 10.4 | 57.0 | 692.1 | 3.0 | 1959-2017 |
| 61 | Juxian | 35.6 | 118.8 | 13.8 | 5.9 | 18.7 | 12.9 | 8.2 | 70.8 | 777.6 | 2.4 | 1990-2017 |
| 62 | Jinghe | 34.4 | 109.0 | 12.6 | 5.2 | 19.9 | 14.7 | 10.6 | 62.6 | 521.1 | 25.0 | 2006-2017 |
| 63 | Huaian | 33.6 | 118.9 | 13.1 | 5.3 | 19.7 | 14.9 | 11.0 | 72.3 | 980.1 | 2.4 | 2001-2017 |
| 64 | Haikou | 20.0 | 110.3 | 14.0 | 5.7 | 28.1 | 24.2 | 21.5 | 83.3 | 1706.5 | 2.8 | 1957-2017 |
| 65 | Hailar | 49.3 | 119.7 | 13.9 | 7.3 | 5.6 | -0.8 | -6.6 | 66.3 | 353.5 | 3.2 | 1960-2017 |
| 66 | Suolun | 46.6 | 121.2 | 14.7 | 7.7 | 10.6 | 3.0 | -3.5 | 56.8 | 447.3 | 2.8 | 1992-2017 |
| 67 | Altay | 47.7 | 88.1 | 15.2 | 8.2 | 10.9 | 4.6 | -1.2 | 58.1 | 197.8 | 2.3 | 1960-2017 |
| 68 | Tacheng | 46.7 | 83.0 | 15.2 | 7.9 | 14.8 | 8.0 | 2.3 | 57.4 | 304.8 | 2.2 | 1993-2017 |
| 69 | Yandie | 42.1 | 86.6 | 15.4 | 8.1 | 16.6 | 9.1 | 2.4 | 58.2 | 79.9 | 1.6 | 1993-2017 |
| 70 | Turpan | 42.9 | 89.2 | 15.4 | 7.9 | 21.8 | 14.8 | 8.6 | 39.6 | 15.0 | 1.2 | 1960-2017 |
| 71 | Kashi | 39.5 | 75.8 | 15.7 | 7.7 | 18.4 | 12.1 | 6.0 | 50.1 | 70.4 | 18.0 | 1957-2017 |
| 72 | Hetian | 37.1 | 79.9 | 16.2 | 7.2 | 19.3 | 12.9 | 7.3 | 41.2 | 39.1 | 19.0 | 1957-2017 |
| 73 | Hami | 42.8 | 93.5 | 17.1 | 9.2 | 18.1 | 10.1 | 3.1 | 43.4 | 39.8 | 1.8 | 1961-2017 |
| 74 | Jiuquan | 39.8 | 98.5 | 16.6 | 8.4 | 15.4 | 8.1 | 1.6 | 46.9 | 90.7 | 21.0 | 1993-2017 |
| 75 | Minqin | 38.6 | 103.1 | 16.6 | 8.5 | 16.3 | 8.6 | 1.7 | 44.4 | 114.1 | 2.7 | 1957-2017 |
| 76 | Erenhot | 43.6 | 111.9 | 17.3 | 8.7 | 12.0 | 4.4 | -2.2 | 47.2 | 135.2 | 4.0 | 1957-2017 |
| 77 | Hohhot | 40.9 | 111.6 | 16.5 | 8.3 | 12.2 | 5.1 | -1.1 | 54.5 | 347.1 | 17.0 | 1959-1968 |
| 78 | Dongsheng | 39.8 | 110.0 | 16.1 | 8.3 | 12.7 | 7.2 | 2.6 | 47.5 | 380.3 | 2.8 | 1992-2017 |
| 79 | Yinchuan | 38.5 | 106.2 | 16.4 | 7.9 | 16.3 | 9.5 | 3.6 | 55.2 | 198.7 | 2.0 | 1959-2017 |
| 80 | Xilinhot | 44.0 | 116.1 | 15.4 | 8.1 | 10.3 | 3.2 | -3.0 | 55.7 | 277.6 | 3.3 | 1990-2017 |

### 2.3.3 Detailed description of four apple phenology models

It is currently argued that photoperiod influences the forcing rate of trees. Hence, building upon the classical Thermal Time model, a novel model named M1 (Equations 1-2 in Table 3, the same below) is proposed, where photoperiod is explicitly included as a driving factor. This model extends the traditional Thermal Time model by incorporating an additional exponential constant. Different from the M1 model, the Uniforc model used sigmoid response function for forcing accumulates (Equations 3-4). The Alternating model integrates both forcing and chilling requirements (Equations 5-6). Unlike traditional models where temperature contributes solely to forcing accumulation, in the Alternating model, each day can contribute to either forcing or chilling accumulation based on the daily mean temperature. In the Alternating model, specific temperature thresholds are defined to determine whether a day contributes to forcing or chilling accumulation. If the daily mean temperature exceeds a certain threshold, that day contributes to forcing accumulation. Conversely, if the temperature falls below another threshold, the day contributes to chilling accumulation. Days falling between these thresholds do not contribute to either accumulation. In Unichill model, the forcing accumulation is the sigmoid function, and the chilling requirement responds to the bell-shaped curve function (Equations 7-10). By integrating forcing and chilling requirements, the Unichill model comprehensively explains how temperature dynamics drive the timing of key apples' developmental stages.

**Result** **of Spatiotemporal changing trends of the effective precipitation**

Changes in accumulated effective precipitation (*P_eff_*) during apples' phenological stages significantly influence irrigation water consumption and scheduling. Quantifying the distribution and changes of effective precipitation can help adjust irrigation arrangements according to actual apple water needs to ensure reasonable water supply for apples. The cumulative effective precipitation across apple-producing counties in China during various phenological stages was illustrated in Fig. S1. Among apple's three phenological phases, the fruit expansion stage had the highest cumulative precipitation, ranging from 24 to 419 mm, with a multi-year average of 215 mm. The cumulative effective precipitation ranged from 4 to 106 mm during the flowering-fruit setting stage and from 6 to 288 mm during the coloring-maturing stage of apples. The multi-year average cumulative effective precipitation during the apple flowering-fruit setting stage (31 mm) is lower than that of the apple coloring-maturing stage (76 mm). Before 1970, the annual cumulative effective precipitation was higher than the multi-year average level during the apple flowering-fruit setting stage (Fig. S1a). However, there was a decrease in the annual cumulative effective precipitation of this phenological stage from 1970 to 1980 and again from 1995 to 2000. After 2000, the annual cumulative effective precipitation of apple flowering-fruit setting stage fluctuated slightly around the multi-year average. The cumulative effective precipitation during the apple fruit expansion period from 1968 to 1980 and 1995 to 2000 was lower than the multi-year average (Fig. S1b), following a trend similar to that of the flowering-fruit setting stage. After 2000, the increasing tendency of cumulative effective precipitation during this stage was observed. The cumulative effective precipitation exhibited differential fluctuations during the coloring-maturing stage, characterized by instances of both extremely high and low precipitation in individual years (Fig. S1c).


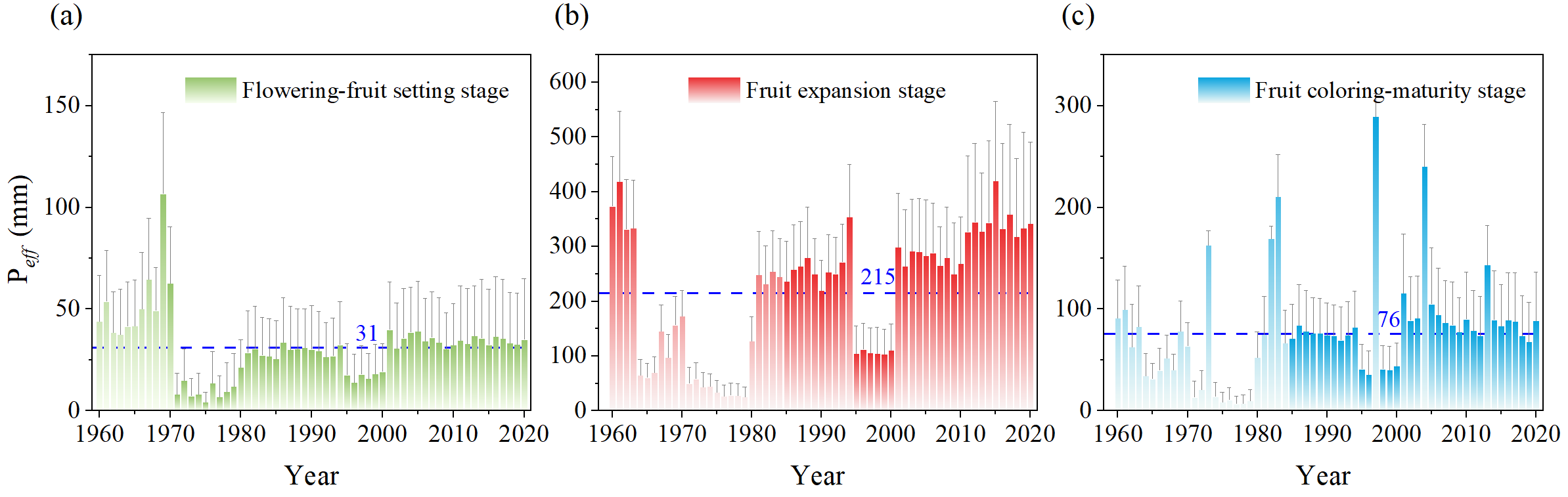


Fig. S1 Temporal variation of accumulated effective precipitation (*P_eff_*) in apple flowering to fruit-setting stage (a), fruit expansion stage (b), and fruit coloring maturity stage (c) from 1960 to 2020 in the whole apple-producing areas of China (The blue dotted line is the average line)

The spatial distribution and frequency histogram of cumulative effective precipitation in major apple-producing areas across the country are displayed (Fig. S2). The cumulative effective precipitation during the apple flowering-fruit setting stages ranged from 4 to 217 mm in different apple-producing counties, with an average of 51 mm. The cumulative effective precipitation during the apple fruit expansion stage ranged from 24 to 703 mm across different apple-producing counties, while during the coloring-maturity stage, it ranged from 6 to 296 mm. On average, the cumulative effective precipitation during the apple fruit expansion stage was 335 mm, and during the coloring-maturity stage, it was 110 mm across different apple-producing counties.


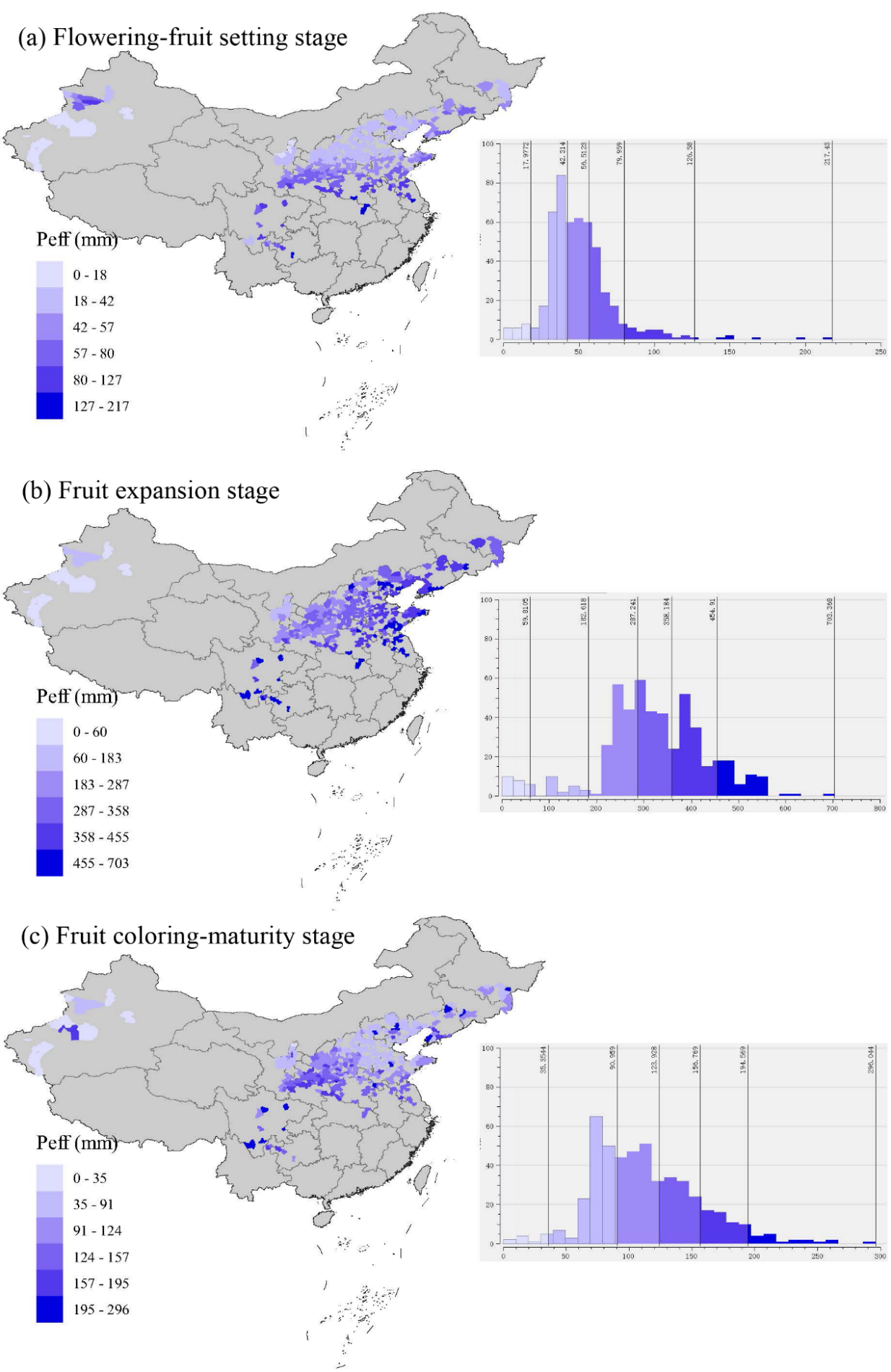


Fig. S2 Spatial distribution map and frequency histogram of accumulated effective precipitation (*P_eff_*) during apple flowering-fruit setting stage (a), fruit expansion stage (b), and fruit coloring-maturity stage (c) in the whole apple-producing areas of China (The blue dotted line is the average line)
